# Toward an Accurate Black-Box Tool for the Kinetics of Gas-Phase Reactions Involving Barrier-less Elementary Steps

**DOI:** 10.1021/acs.jctc.3c00857

**Published:** 2023-10-26

**Authors:** Luigi Crisci, Silvia Di Grande, Carlo Cavallotti, Vincenzo Barone

**Affiliations:** † Scuola Normale Superiore di Pisa, Piazza dei Cavalieri 7, I-56126 Pisa, Italy; ‡ Department of Chemical Sciences, University of Napoli Federico II, Complesso Universitario di M.S. Angelo, via Cintia 21, I-80126 Napoli, Italy; § Scuola Superiore Meridionale, Largo San Marcellino 10, I-80138 Napoli, Italy; ∥ Department of Chemistry, Materials and Chemical Engineering “G. Natta”, Politecnico di Milano, I-20131 Milano, Italy

## Abstract

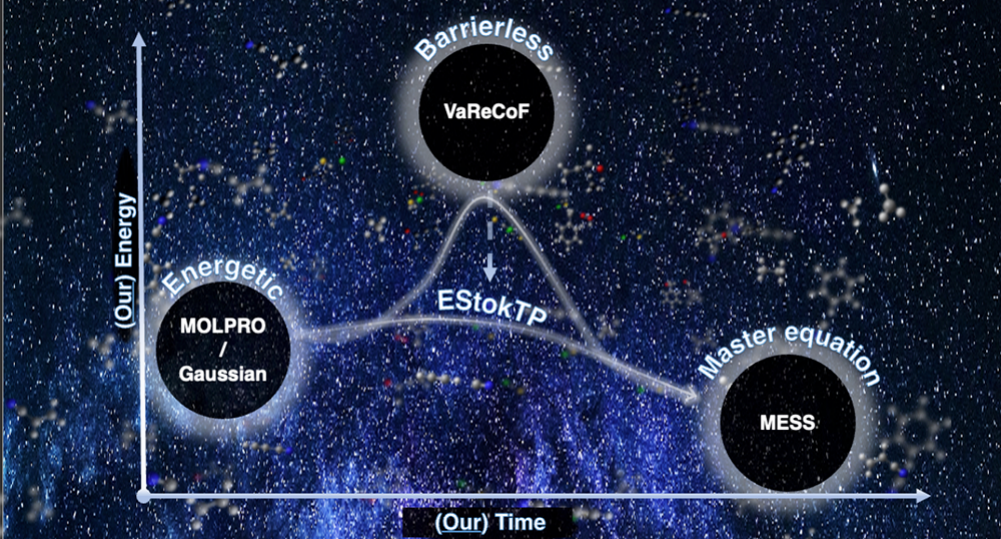

An enhanced computational protocol has been devised for the accurate characterization of gas-phase barrier-less reactions in the framework of the reaction-path (RP) and variable reaction coordinate variational transition-state theory. In particular, the synergistic combination of density functional theory and Monte Carlo sampling to optimize reactive fluxes led to a reliable yet effective computational workflow. A black-box strategy has been developed for selecting the most suited density functional with reference to a high-level one-dimensional reference potential. At the same time, different descriptions of hindered rotations are automatically selected, depending on the corresponding harmonic frequencies along the RP. The performance of the new tool is investigated by means of two prototypical reactions involving different degrees of static and dynamic correlation, namely, H_2_S + Cl and CH_3_ + CH_3_. The remarkable agreement of the computed kinetic parameters with the available experimental data confirms the accuracy and robustness of the proposed approach. Together with their intrinsic interest, these results also pave the way toward systematic investigations of gas-phase reactions involving barrier-less elementary steps by a reliable, user-friendly tool, which can be confidently used also by nonspecialists.

## Introduction

The prediction and interpretation of rate constants for gas-phase reactions play a fundamental role in different research areas such as combustion,^1–5^ atmospheric chemistry,^6–9^ and astrochemistry.^10–13^ Recent advances in hardware, software, and, above all, the underlying quantum chemical approaches are providing results whose accuracy can rival that of the most sophisticated experimental studies, at least for elementary processes involving small, semirigid molecules and distinct energy barriers.^14–17^ In this connection, transition state (TS) theory (TST)^18,19^ still plays a central role, with the Rice–Ramsperger–Kassel–Marcus theory^20–26^ allowing for the calculation of rate constants in a microcanonical ensemble using a limited number of points on the target reactive potential energy surfaces (PESs). The main limitations of this approach are related to some intrinsic weaknesses of the semiclassical TST formulation,^27,28^ including the treatment of quantum effects on the reaction coordinate (e.g., tunneling and nonclassical reflection^29,30^) or the nonrecrossing assumption.^31–33^ Corrective scaling factors^34^ can be used to address the former issue, while variational TST (VTST)^35–39^ tackles the latter issue by minimizing the reactive flux as a variational variable, thus defining a generalized TS at a dynamical bottleneck.^38,40^ The accuracy of the model, which is rooted in the TS/reaction coordinate definition, is strongly dependent on the accurate determination of the dividing surface (and so the reaction coordinate) between reactants and products.^41^ In fact, as clearly stated by Marcus in the III paper of the “Analytical Mechanics of Chemical Reactions”^42–44^ series, the same set of coordinates should be able to “pass smoothly from those suited to reactants, through those suited to “activated complexes,” to those suited to products”. The variational principle can be used to obtain the best dividing surface perpendicular to the chosen reaction coordinate by minimizing the reactive flux, with this approach defining a robust and predictive model irrespective of the method used for taking quantum effects into account and for performing density of state counts (e.g., the Stein-Rabinovitch algorithm,^45,46^ the Wang–Landau,^47–49^ or the Monte Carlo (MC) phase-space sampling^50–54^).

In this context, the intrinsic reaction path (RP) and the corresponding Hamiltonian^55^ provide a very convenient framework and are at the core of the RP–VTST model,^18,30,38^ which has become the de facto standard for reactions ruled by distinct energy barriers. The development of effective treatments of tunneling and nonclassical reflection effects [e.g., the small curvature tunneling (SCT) variant^56^ employed in the present study] has further widened the field of application of TST. Further progress has been made by the replacement of Cartesian coordinates with curvilinear internal coordinates in the treatment of motions orthogonal to the reaction coordinate.^57^ Finally, multistructural VTST, which is a version of RP–VTST that uses several RPs, does optimize the shape of the dividing surface for the specific case of torsional modes.^58^ In fact, large-amplitude motions (e.g., hindered rotations, inversions, ring puckerings, etc.) need special care.^59–65^ Here, we address this issue by enforcing a smooth transition between different descriptions of those degrees of freedom depending on their harmonic frequencies along the RP. As we will see, this approach can significantly improve the computed global rate constants without any increase in computational requirements.

A different variant of VTST, referred to as variable reaction coordinate (VRC), has been introduced to properly address barrier-less reactions,^66,67^ which occur in various contexts, such as initial steps of complex reaction schemes^68,69^ in interstellar molecular clouds, whose extreme conditions lead to gas-phase reactions predominantly driven by radical or ionic species.^70–72^ The same issue is encountered in combustion and atmospheric chemistry, where bond cleavage in molecules like hydrocarbons generates radicals, triggering chain reactions that form soot and other pollutants^73–75^ closely related to atmospheric pollution issues.^76–78^

As the name implies, VRC–VTST introduces a way to define a reaction coordinate that changes with the interfragment separation, going from the distance between the nearest atoms involved in the reaction belonging to different fragments when approaching new bond formation to the distance between fragment centers-of-mass when approaching the reactant asymptote.^79–81^

The theoretical framework employed in the present context combines RP–VTST and VRC–VTST, with the latter being based on classical phase-space MC sampling to minimize fluxes. This approach leads to an effective definition of the most suitable dividing surface by considering only the numbers of states for the transitional modes, i.e., the large-amplitude motions, whose nature changes during the approach of the reactants.^82^

While an accurate electronic structure method would be required to perform the MC sampling, the use of a cheap level of theory becomes unavoidable in view of the huge number of points required to obtain well-converged results. An effective solution to this dilemma is implemented in the VaReCoF code^66,67,83,84^ and is based on the on-the-fly correction of the results obtained by the MC sampling with a low-level (LL) method by means of the difference between high-level (HL) and LL results for a one-dimensional cut of the PES, the so-called reference potential.^85–90^ In particular, the HL method is usually a (large basis set)/(large active space) multireference computation [typically CAS-SCF/CAS(PT2)], whereas the LL method is the corresponding (medium basis set)/(small active space) method.^85–92^

The main limitation of this approach is that the LL method can badly describe the correct wave function in some regions of the PES.^93–95^ Furthermore, it has been observed that an orientation-independent corrective potential and geometry relaxation might be inadequate, and, more generally, the geometry relaxation correction conflicts with the assumption that the partition functions of conserved modes cancel between reactants and generalized TSs.^96^ Since some modern density functionals are expected to accurately describe both van der Waals interactions and static correlation effects, a VRC–VTST approach employing rigid geometry MC samplings with those functionals has been implemented in the Polyrate code.^97^

In our opinion, a general strategy (followed in the present work) can be based on the combination of flexible and robust density functional theory (DFT) models for MC sampling, with the use of one-dimensional HL potentials. These latter potentials can be used for selecting the most suited DFT/basis set, depending on the target systems, and to correct its results. Furthermore, the consistency between different points of the PES can be improved by employing the active space and/or density matrix of previous points as an initial guess for the current one.

Since the refinement of a corrective potential might be a laborious and time-consuming procedure, we have devised a novel protocol that applies the aforementioned concepts in a black-box fashion. To this end, a user-friendly code has been built in order to interface an EStokTP^98^ development version with the VaReCoF code^84^ for the automatic determination of the DFT level of theory that best fits a reference potential.

The overall workflow, including all the improvements sketched above, has been tested for two types of barrier-less reactions that differ in their inherent multireference character, namely, a hydrogen abstraction (H_2_S + Cl → HS + HCl) and a radical–radical association (CH_3_ + CH_3_ → C_2_H_6_). Furthermore, the two processes are prototypical examples of a multistep radical–molecule addition elimination and a single-step radical–radical addition reaction.

Besides representing a challenging case study, the H_2_S + Cl reaction plays an important role in atmospheric chemistry, being related to acid rains, visibility reduction, and climate change.^99–103^ The Earth’s stratosphere is enriched with hydrogen sulfide, mainly removed by the hydroxyl radical,^104^ from volcanic eruptions and organic decomposition.^105,106^ However, in some marine remote boundary layers and coastal metropolitan zones, the chlorine radical concentration is larger than that of the hydroxyl radical,^107^ making the interaction between H_2_S and Cl equally crucial.

On the other hand, the CH_3_ + CH_3_ → C_2_H_6_ case study is important in combustion chemistry as a termination reaction, as a source of C_2_ species in methane oxidation,^76^ and in planetary atmospheres following methane photolysis.^108,109^ Moreover, this reaction gives us the opportunity to investigate radical–radical barrier-less combinations that play a significant role at low temperatures in atmospheric and interstellar chemistry.

A last motivation for the selection of these two reactions is that both of them are well characterized from experimental^110–125^ and theoretical^80,96,126–135^ standpoints.

## Methods

### Reference Structures and Vibrational Frequencies

Geometry optimizations and evaluations of harmonic and anharmonic force constants were carried out by using modern hybrid and double-hybrid density functionals, which deliver remarkably accurate structural and spectroscopic properties.^136–143^ In particular, the rev-DSDPBEP86 double-hybrid density functional^144^ was used in conjunction with the jun-cc-pVTZ basis set.^145–147^ Empirical dispersion corrections were added by means of Grimme’s D3 scheme^148^ with Becke-Johnson damping [D3(BJ)].^149^ Since tight *d* functions are important for a quantitative representation of the electronic structure of second-row elements,^150^ partially augmented basis sets, namely, jun-cc-pV(*n* + *d*)Z (hereafter j*n*) with *n* = *T*, *Q*, including an additional set of tight *d* functions for sulfur and chlorine atoms, were employed. The overall computational model (density functional and basis set) will be denoted in the following as rDSD. During geometry optimizations, custom grid values were employed, resulting in a finer grid than the Gaussian’s Int = UltraFine one [reproducible by using int = (grid = 199974) in the Gaussian route section]. After full geometry optimization, analytical Hessians were computed and employed to obtain by numerical differentiation the cubic and semidiagonal quartic force constants needed for the evaluation of anharmonic zero-point energies (ZPEs) in the framework of vibrational perturbation theory to second-order.^151–154^ In particular, resonance-free expressions have been employed for both the ZPE^60,155^ and the vibrational frequencies.^60,64,156^

All the computations were performed by the Gaussian16 suite of programs^157^ using the unrestricted formalism for open shells.

### Energetics

Improved electronic energies were obtained by single-point energy evaluations with accurate wave function methods at rDSD geometries.

For systems not exhibiting strong multireference character, the coupled cluster (CC) model, including single, double, and perturbative estimates of triple excitations [CCSD(T)],^158^ is known to deliver accurate electronic energies, provided that complete basis set (CBS) extrapolation and core–valence (CV) correlation are taken into proper account. In this framework, the family of reduced-cost “Cheap” (ChS) schemes offers a good compromise between cost and accuracy.^159–164^ In particular, the latest (and most reliable) member of this family (junChS-F12) employs the following expression^162,164^
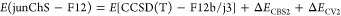
1where the CBS extrapolation is performed using MP2-F12 energies and the *n*^–3^ formula^165^
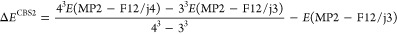
2and the contribution of CV correlation is estimated by the MP2-F12 energy difference between all-electron (ae) and frozen core (fc) calculations employing the cc-pwCVTZ^166,167^ (wC3) basis set
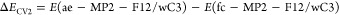
3

For the purpose of validation, additional terms can be added to obtain higher-accuracy results
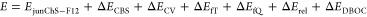
4

The CBS and CV contributions refer to the differences between the evaluations of these terms at the CCSD(T)-F12 and MP2-F12 levels. The diagonal Born–Oppenheimer correction Δ*E*_DBOC_^168–171^ and the scalar relativistic contribution to the energy Δ*E*_rel_^172,173^ are computed at the HF-SCF/aug-cc-pVDZ^147^ and CCSD(T)/aug-cc-pCVDZ^166^ level. Finally, the corrections due to full treatment of triple (Δ*E*_fT_) and quadruple (Δ*E*_fQ_) excitations are computed, within the fc approximation, as energy differences between CCSDT and CCSD(T) and between CCSDTQ and CCSDT calculations employing the cc-pVTZ and cc-pVDZ basis set,^174^ respectively.

For the ethane system, a full-valence complete active space including 14 electrons in 14 orbitals (14e, 14o) was treated at the CAS-SCF/CAS(PT2)/aug-cc-pVQZ level of theory. In fact, this approach provides a robust description of the multireference character inherent in the ethane association, at variance with the hydrogen abstraction process.

The MOLPRO software^175–177^ was used for all computations except CCSDT and CCSDTQ ones, which were performed by the MRCC code.^178^

### Kinetic Model

VRC–VTST simulations were performed using the VaReCoF software, while master equation results were obtained with the MESS program.^179,180^ The determination of stationary points to select the most effective potential for VRC–VTST simulations, VTST in internal curvilinear coordinates,^57^ and the input files for both VaReCoF and MESS codes have been produced by a development version of the EStokTP program. As mentioned above, several modifications have been made to increase the effectiveness and flexibility of the MC sampling and the one-dimensional corrective potential. The master equation simulations have been performed by the MESS program and employing a single exponential down model with a temperature dependence Δ*E*_down_ of 260(T/298)^0.875^ cm^–1^ for describing the probability of collisional energy transfer.^181^

### H_2_S + Cl

**Figure 1:**
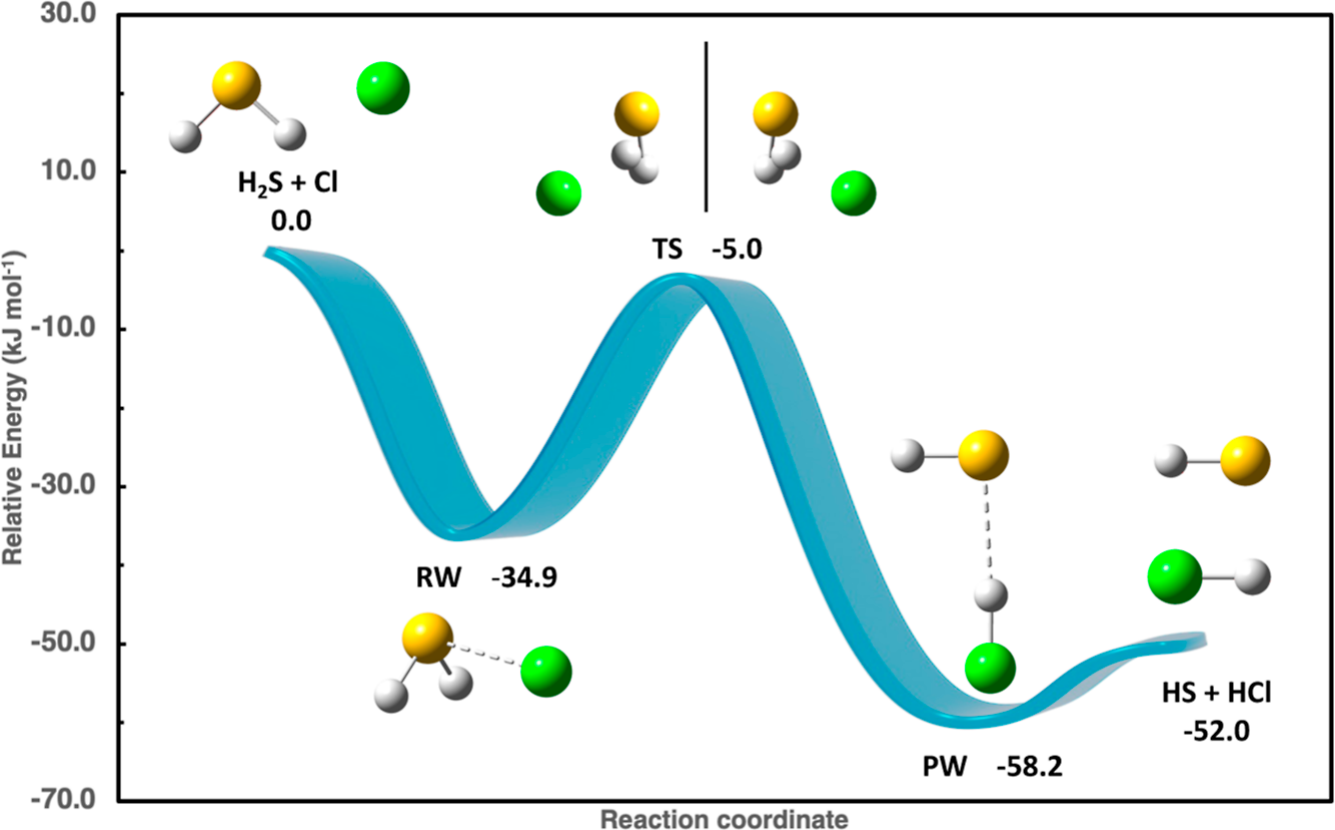
Reaction mechanism for H_2_S + Cl reaction, employing junChS-F12 energies including anharmonic ZPEs (kJ mol^–1^).

The H_2_S + Cl reaction mechanism involves the sequence of elementary steps shown in Figure 1: the initial interaction between hydrogen sulfide (H_2_S) and chlorine radical (Cl), ruled by noncovalent forces, leads to a prereactive complex (RW). Then, under atmospheric conditions, the only open reaction channel follows an addition/elimination mechanism.

After the isomerization of the RW complex governed by the TS, the reaction proceeds toward the final van der Waals complex (PW), stabilized by a hydrogen bond between the HS and HCl moieties. Dissociation of the PW complex gives the final products of hydrogen abstraction, HS radical, and HCl. Therefore, the whole reaction can be described using a three-step, two-well master equation. The overall rate constant is tuned by temperature and pressure, which determine the variable role played by the conversion rate of RW into PW and the formation rate of RW. The decomposition rate of PW plays a negligible role since it is faster than the other two steps. In any case, the formation of the prereactive complex, through a barrier-less step, plays a key role. The computational protocol described above has been applied starting from a detailed exploration of the PES, parametrized as a function of the internuclear distance between Cl and S in the 3.0–4.6 Å range, with a step of 0.2 Å. For each point, the other geometrical parameters have been optimized at the rDSD level, and the HL reference potential has been obtained by single-point junChS-F12 energy evaluations for these geometries. The values of the T_1_ diagnostic and spin contamination shown in Figure S11 of the Supporting Information confirm the reliability of the JunChs-F12 single-reference model in the region of interest to describe the outer TS. In the case of ethane dissociation, the same diagnostic *T*_1_ reaches the unacceptably large value of 0.03 for an interfragment separation of 3.2 Å, thus suggesting that a multireference approach is necessary in that case.

A key feature of MC sampling is the application of a distance-dependent corrective potential to the sampled energies. In this regard, the first step of the new workflow involves automated single-point calculations employing user-defined levels of theory for all of the previously determined stationary points. In the present case, a panel of density functionals (B2PLYP, B3LYP, BLYP, CAM-B3LYP, MPWPW91, and PBE0)^182–186^ and basis sets [6-31G(d,p) and 6-31G(3df,2p), cc-pVTZ and cc-pVQZ possibly including also diffuse functions]^174,187–190^ have been tested in conjunction with counterpoise corrections for the basis set superposition error (BSSE).^191,192^ Next, these single-point calculations are employed to derive the corrective potentials with respect to the reference HL potential.

**Figure 2:**
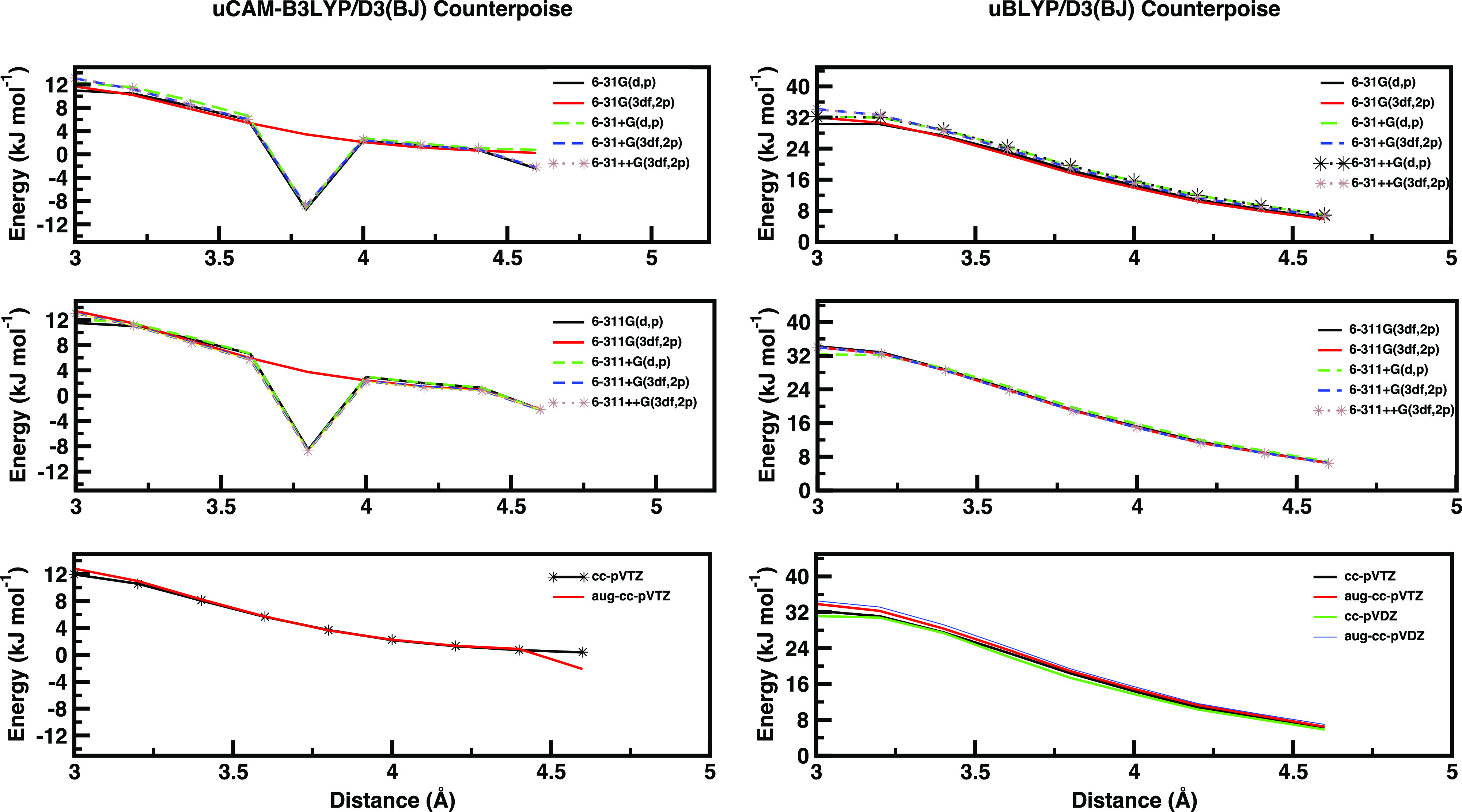
H_2_S + Cl barrier-less entrance channel HL corrective potentials comparison between uCAM-B3LYP and uB3LYP with counterpoise corrections.

**Figure 3:**
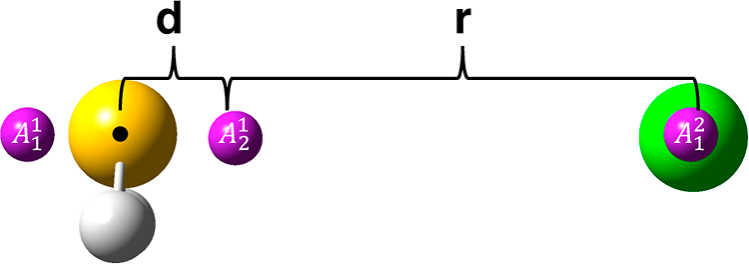
Pivot points and *d* and *r* parameters are used to build the multifaceted dividing surfaces for the H_2_S + Cl reaction.

For purposes of illustration, CAM-B3LYP and BLYP results are shown in Figure 2, whereas the full set of results is shown in Figures S1–S10 of the Supporting Information. Some combinations of functional (including CAM-B3LYP) and basis sets show numerical instabilities for interfragment distances between 3.0 and 4.0 Å and are, therefore, excluded from the candidates for full MC sampling. As can be deduced from Figure 2, the corrective potential is remarkably dependent on both the DFT exchange–correlation functional and basis set. A more detailed analysis of the different behavior shown by the investigated exchange–correlation functionals is beyond the purposes of the present paper. Once the DFT protocol and the corrective potentials have been determined, the EStoKP machinery is used to generate a suitable input for VaReCoF, which performs the MC sampling over multifaceted dividing surfaces. In the present case, the dividing surfaces are built with the help of the three pivot points shown in Figure 3.

Two pivot points (A_1_^1^ and A_2_^1^) are placed near the S atom, symmetrically displaced along the axis perpendicular to the H_2_S plane and at variable distance *d* from S. The third pivot point, A_1_^2^, is centered on Cl. The multifaceted dividing surface is then built by varying the Cl–pivot distance in the interval between 5 and 11 a_0_ with a step of 1.0 a_0_.^67^ Furthermore, the calculations have been repeated by changing the position of the A^1^ pivot points, with variations of the parameter *d* between 0.01 and 0.6 a_0_, with a 0.1 a_0_ step.

The MC sampling convergence threshold for the reactive flux computation is set to 5%, and a minimum of 200 points have been taken for each dividing surface. A flat 0.9 factor is employed to account for recrossing dynamical effects. This factor was derived from a number of test computations,^67^ which showed an average overestimation of 10% for the VRC–VTST total rate coefficients with respect to reference trajectory simulations.

The elementary step leading from RW to PW is governed by a distinct energy barrier along a path involving the motion of a hydrogen atom, thereby highlighting the necessity of accounting for quantum effects along the reaction coordinate. This step can be effectively treated in the framework of VTST with SCT.^56^ Therefore, the calculations of structures and frequencies along the intrinsic reaction coordinate play a central role. The IRC has been characterized at the rDSD level by 100 steps of 0.03 au, starting from the TS in both the reactant and product directions. The harmonic frequencies along the IRC have been computed again at the rDSD level in both Cartesian and internal curvilinear coordinates. Those data are next employed to compute SCT corrections.

The first-order saddle point connecting PW and RW is characterized by two optical isomers (Figure 1), which interchange through a large-amplitude motion involving the Cl atom and evolve through a second-order saddle point in which the Cl atom lies in the H_2_S plane. This feature can be properly addressed by a one-dimensional hindered rotor potential, which has been determined by performing a relaxed scan and computing the vibrational frequencies at each point. However, near the second-order saddle point, geometry optimizations suffer from strong numerical instability problems. As a consequence, after full optimization of the geometry of the second-order saddle point, we use these data together with those of the first-order saddle point to fit a sinusoidal function, which provides the input energies for the MESS treatment of a single hindered rotor. An additional issue is related to the transitional nature of this large-amplitude motion along the IRC leading from TS to RW or PW. In fact, the use of a single model (either hindered rotor or harmonic oscillator) along the whole IRC could lead to temperature-dependent errors on the final rate constant. To circumvent this problem, the same hindered rotor treatment at the TS and at points on the IRC close to it is employed. To check the stability of the results, we compared two different strategies. In the first approach, the harmonic oscillator is swapped with the hindered rotor exclusively for those points on the IRC where the computed harmonic frequency for this mode is within a narrow range around the value at the TS. This approach (named the hindered cutoff) led to the application of the hindered rotor model to a specific set of 44 IRC points. In the second, more drastic approach (defined as hindered on TS), the hindered rotor model is employed only for the two points (one on each side) closest to the TS.

### CH_3_ + CH_3_

In this case, since the reaction consists of a single barrier-less step, attention has been focused on the multifaceted dividing surface description and the selection of the most suitable corrective potential. Only the singlet PES has been investigated since, as already pointed out by Klippenstein and Harding,^193^ the contribution of the triplet state is negligible due to ineffective intersystem crossing.

The corrective potential is obtained from the sum of the already described rigid geometry corrective potential and a geometric one. The latter potential is obtained from the difference between the potential energy of the optimized structures at different interfragment separations and the corresponding ones in which conserved modes are frozen. As expected, the weight of the geometric corrective potential is higher at short C–C distances and smoothly decays at large separations between the two methyl radicals. The functionals tested to minimize the corrective potential include BLYP, B3LYP, CAM-B3LYP, B2PLYP, M06, and M06-2X^194^ with and without counterpoise corrections, in conjunction with the same basis sets discussed in the case of H_2_S + Cl. The complete results of the systematic study (with and without counterpoise corrections) are collected in Tables S1–S6 of the Supporting Information, whereas the most significant results are summarized in Table 1. It is quite apparent that the role of the basis set is less important than that of the functional form, with the BLYP/6-31++G(d,p) model chemistry offering the best compromise between accuracy and computational cost. As expected, GGA functionals are more reliable than their hybrid or even double-hybrid counterparts in the presence of strong static correlation effects. Thus, the remarkable difference between the two reactions examined in the present work points out the advantages of an automatic, system-dependent selection of the most suitable cheap model chemistry to be employed in MC sampling.

**Table 1:** Maximum, Minimum, and Average Corrective Potential Relative Deviations for the Ethane System^a^

level of theory	max relative deviation (%)	min relative deviation (%)	average relative deviation (%)
uB2PLYP/6-311+G(3df,2p)	55	37	47
uB2PLYP/6-31G(d,p)	73	34	45
uB2PLYP/aug-cc-pVQZ	53	37	46
uB3LYP/6-311+G(3df,2p)	43	25	33
uB3LYP/6-31G(d,p)	46	14	26
uB3LYP/aug-cc-pVQZ	42	24	33
uBLYP/6-311+G(3df,2p)	40	1	12
uBLYP/6-31++G(d,p)	39	0	11
uBLYP/6-31G(3df,2p)	37	3	20
uBLYP/6-31G(d,p)	36	4	14
uCAM-b3lyp/6-311G(3df,2p)	51	7	37
uCAM-b3lyp/6-31G(d,p)	56	24	37
uCAM-b3lyp/aug-cc-pVQZ	50	34	44
uM06-2*X*/6-311+G(3df,2p)	68	6	35
uM06-2*X*/6-31G(d,p)	50	6	28
uM06-2X/aug-cc-pVDZ	64	3	35
uM06/6-311G(3df,2p)	40	3	23
uM06/6-31G(d,p)	40	14	27

a All computations were performed applying D3(BJ) semi-empirical corrections.

**Figure 4:**
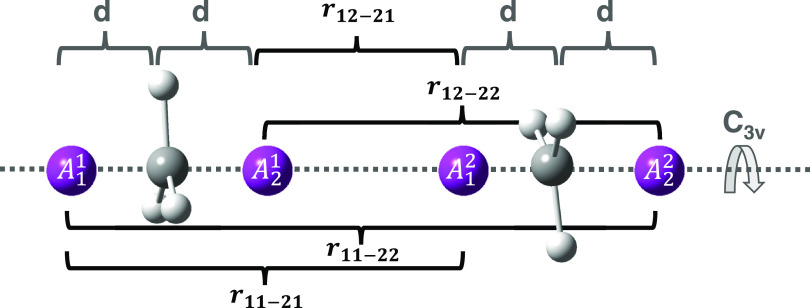
Pivot points and *d* and *r* parameters used to build the multifaceted dividing surfaces for the CH_3_ + CH_3_ reaction.

Next, the dividing surfaces are generated by placing two couples of pivot points ({*A*_1_^1^–*A*_2_^1^} and {*A*_1_^2^–*A*_2_^2^}, which respectively belong to fragments one and two), one for each CH_3_ side, along the *C*_3*v*_ axis of each fragment, taking into account all kinds of recombination processes (Figure 4).

The sampling is performed by building a total of 27 dividing surfaces. Three initial configurations are considered. The distance *d* between each pivot point couple and the center of mass of the fragment to which it belongs is altered from a value of 0.01 to 0.3 and 0.5 *a*_0_ first. For each configuration, the sampling is performed by changing four parameters (i.e., the distances between each pivot point of a given fragment and the other fragment ones, labeled as *r*_11–22_, *r*_11–21_, *r*_12–21_, and *r*_12–22_ in Figure 4) in the interval [4.5, 8.5] *a*_0_ with a grid spacing of 0.5 *a*_0_.

## Results and Discussion

### Stationary Points of H_2_S + Cl

The results of a systematic study of basis set and method effects on the relative energies of the stationary points ruling the H_2_S + Cl reaction are collected in Tables S7 and S8 of the Supporting Information, whereas the most significant results are summarized in Table 2. While the recent comprehensive study by Lupi et al.^126^ provided quite accurate results, it is apparent that further basis set extension has a non-negligible effect, especially on the energy barrier (TS). When pushing the basis sets to the limit of available compilations, conventional and explicitly correlated^195^ (F12) approaches provide comparable results (within 0.5 kJ mol^–1^). Furthermore, the role of triple and quadruple excitations is quite limited, whereas the CV correlation cannot be neglected to obtain quantitative results. Remarkably, the difference between harmonic and anharmonic ZPEs provides contributions of comparable magnitude. Finally, spin–orbit (SO) contributions are not negligible only for reactants (3.3 kJ mol^–1^) and products (2.1 kJ mol^–1^). In our previous study of the H_2_S + Cl reaction,^126^ SO contributions were calculated along the RP using the Breit–Pauli Hamiltonian and CAS-SCF wave functions. It was found that starting from 4.2 Å, they contribute less than 1 kJ mol^–1^ to the long-range potential, which was considered to be an acceptable error for the VRC–TST calculations. Also, at the submerged saddle point, the SO splitting essentially vanishes. It was thus concluded that just correcting the asymptotic energy of the reactants for SO was sufficient for the intended accuracy of the present approach.

**Table 2:** Relative Electronic Energies for the Stationary Points of the H_2_S + Cl Reaction Were Obtained by Different Methods^a^

	reactants	RW	TS	PW	products
level of theory	H_2_S + Cl	H_2_S···Cl	HS···H···Cl	HS···HCl	HS + HCl
CBS(4Z, 5Z)	0.0	–44.8	–3.1	–60.1	–47.5
CBS(a5Z, a6Z)	0.0	–44.2	–2.1	–59.5	–47.2
CBS(a3F12, a4F12)	0.0	–44.8	–2.5	–60.0	–47.5
heat-like	0.0	–41.9	0.0	–56.8	–46.2
best conv	0.0	–41.3	1.0	–56.2	–45.9
best F12	0.0	–41.9	0.6	–56.8	–46.2
junChS-F12	0.0	–40.4	0.9	–56.8	–46.4
ΔZPE_h_	0.0	5.9	–5.3	–1.6	–5.9
ΔZPE_a_	0.0	5.5	–5.9	–1.4	–5.6

a CBS means CBS extrapolation, and the basis sets are referred to as *n*Z for cc-pV*n*Z and *n*F12 for cc-pV*n*Z-F12.^196^ The prefix *a* is used for augmented basis sets. Finally, conv. is used for conventional approaches, F12 for their explicitly correlated counterparts, and best for the computations involving all of the additional terms collected in eq 4. rDSD/j3 harmonic (ΔZPE_h_) and anharmonic (ΔZPE_a_) ZPE contributions are also given. All of the data are in kJ mol^–1^.

The junChS-F12 model chemistry performs a remarkable job (errors usually within 0.5 kJ mol^–1^ with respect to the most accurate results) in view of its limited computational cost and lack of empirical parameters. The only non-negligible difference from the most accurate results concerns the relative stability of RW, which is underestimated by about 1.0 kJ mol^–1^.

**Figure 5:**
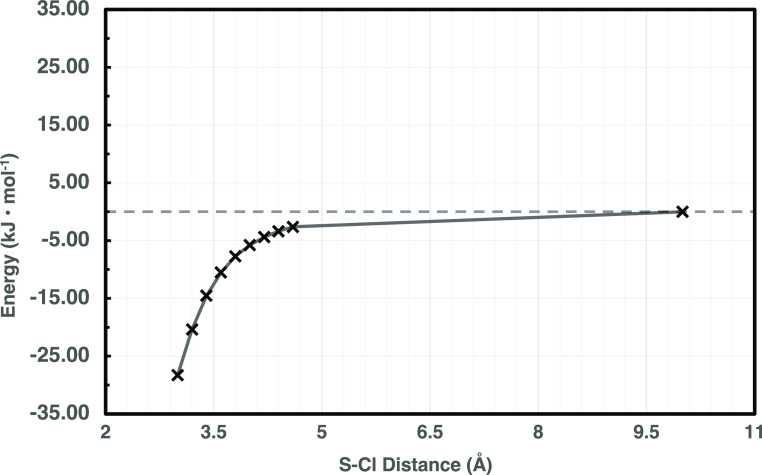
JunChS-F12 potential energy profile (in kJ mol^–1^) as a function of the S–Cl distance (Å). The broken line evidence the reactant asymptote.

Finally, it could be argued that the geometries employed in all of the computations (optimized at the rDSD level) could reduce the overall accuracy of the results. However, optimization of the geometries at the junChS level leads to average differences within 0.002 Å for bond lengths and within 0.2° for angles. Once again, the largest difference is observed for RW (overestimation of 0.01 Å for the S–Cl distance), but the differences in relative energies computed at the junChS-F12 level at rDSD and junChS geometries never exceed 0.2 kJ mol^–1^. In summary, junChS-F12 appears to yield sufficiently accurate results to be used as a HL method in the characterization of the energetics governing the barrier-less entrance channel (Figure 5), and the rDSD model can safely play the same role concerning geometrical structures.

### Rate Constants of H_2_S + Cl

The automatic procedure to select the most suitable level of theory to perform the MC sampling directs our choice to the unrestricted CAM-B3LYP 6-31G(3df,2p) combination of density functional and basis set with counterpoise correction for BSSEs. This computational model shows a maximum difference of 2.8 kJ mol^–1^ for a S–Cl distance of 3.0 Å and an average overestimation of the interaction energy of 1.1 kJ mol^–1^ with respect to the reference potential. The role of the counterpoise corrections is non-negligible, as the maximum and average relative errors increase to 3.3 and 1.5 kJ mol^–1^ without this contribution. In both cases, the corrective potentials decrease smoothly with the interfragment distance toward the asymptotic value of zero.

The temperature dependence of the global rate constants computed by different models is shown in Table 3.

**Table 3:** Three-Step, Two-Well Master Equation Global Rate Constants (cm^3^ molecule^–1^ s^–1^, 1 atm)^a^

	global rate constants			
*T*	expt.^117,119^	only harmonic	hindered on TS	hindered cutoff
202	1.06 × 10^–^^10^	1.18 × 10^–^^10^	1.02 × 10^–^^10^	9.67 × 10^–^^11^
224	8.84 × 10^–^^11^	1.11 × 10^–^^10^	9.46 × 10^–^^11^	8.93 × 10^–^^11^
263	8.35 × 10^–^^11^	1.00 × 10^–^^10^	8.36 × 10^–^^11^	7.91 × 10^–^^11^
290	7.74 × 10^–^^11^	9.42 × 10^–^^11^	7.77 × 10^–^^11^	7.31 × 10^–^^11^
297	7.55 × 10^–^^11^	9.28 × 10^–^^11^	7.63 × 10^–^^11^	7.18 × 10^–^^11^
299	7.37 × 10^–^^11^	9.27 × 10^–^^11^	7.57 × 10^–^^11^	7.16 × 10^–^^11^
355	6.32 × 10^–^^11^	8.33 × 10^–^^11^	6.67 × 10^–^^11^	6.29 × 10^–^^11^
357	6.56 × 10^–^^11^	8.31 × 10^–^^11^	6.64 × 10^–^^11^	6.28 × 10^–^^11^
430	5.92 × 10^–^^11^	7.49 × 10^–^^11^	5.85 × 10^–^^11^	5.51 × 10^–^^11^
433	5.69 × 10^–^^11^	7.47 × 10^–^^11^	5.82 × 10^–^^11^	5.48 × 10^–^^11^
483	5.34 × 10^–^^11^	7.10 × 10^–^^11^	5.41 × 10^–^^11^	5.11 × 10^–^^11^
536	4.77 × 10^–^^11^	6.81 × 10^–^^11^	5.10 × 10^–^^11^	4.82 × 10^–^^11^
610	4.71 × 10^–^^11^	6.54 × 10^–^^11^	4.78 × 10^–^^11^	4.54 × 10^–^^11^
698	4.34 × 10^–^^11^	6.36 × 10^–^^11^	4.54 × 10^–^^11^	4.32 × 10^–^^11^
815	4.14 × 10^–^^11^	6.32 × 10^–^^11^	4.35 × 10^–^^11^	4.15 × 10^–^^11^
914	3.95 × 10^–^^11^	6.40 × 10^–^^11^	4.28 × 10^–^^11^	4.10 × 10^–^^11^

a VRC–VTST for the outer TS, SCT, and RP–VTST for the inner, and phase-space theory for the exit channel. Different models for treating the hindered degrees of freedom in RP–VTST are reported. From left to right: temperature (K), harmonic oscillator model for all steps of IRC (only harmonic), hindered rotor model only on the two nearest points to the TS (hindered on TS), and hindered rotor model on different points of IRC based on a frequency cutoff (hindered cutoff).

The third column of Table 3 reports the results issued from the conventional protocol (full harmonic), which systematically employs the harmonic oscillator model along the whole IRC to treat vibrational degrees of freedom in the RP–VTST formalism. The agreement with the available experimental results is quite satisfactory, with a maximum and average overestimation of 1.62 and 1.33 times, respectively. Nonetheless, the overestimation of the computed rate constants tends to increase with the temperature, going from a factor of 1.11 at 202 K to a factor of 1.62 at 914 K. The incorporation of a hybrid treatment for the large-amplitude/transitional mode leads to a remarkable improvement of the results, irrespective of the specific model employed (hindered on TS or hindered cutoff). Indeed, enforcement of the hindered rotor treatment only at the TS leads to a slight underestimation (3.7%) of the experimental value at 202 K, whereas the largest overestimation (8.8%) is obtained at 914 K, and the average deviation is reduced to a remarkable 3.3%. The hindered cutoff model delivers the same remarkable average error but with an opposite behavior, i.e., an underestimation of 8.8% at 202 K and an overestimation of 3.8% at 914 K.

The accuracy of all the results (reaction rates always within a factor of 1.6 with respect to the experiment) witnesses the reliability of the proposed methods for characterizing reactive PESs, namely, junChS-F12 energies at rDSD optimized geometries for defining a reference one-dimensional potential, together with the new automated selection of the best DFT model for performing the MC sampling. The slight overestimation of the computed results at high temperatures is surely related to the difficulty of describing in a consistent way the density of states related to interconversion between optical isomers in the neighborhood of the inner TS. In this connection, the passage from a hindered rotor to a harmonic oscillator along the IRC plays a significant role in tuning the temperature dependence of the rate constant.

### Energetics of CH_3_ + CH_3_

The same general strategy described for H_2_S + Cl was used in the case of ethane. The main geometrical parameters of the reactant and the products obtained by different computational methods are compared in Table 4 with the available experimental results.

**Table 4:** C_2_H_6_ (Reactant) and CH_3_ (Asymptote) Structural Parameters^a^

		CAS-SCF(10e,10o)//CAS(PT2)/ANO-L^197^	rDSD/aug-cc-pVTZ	exp^198^
Reactant (C_2_H_6_)				
	r_*C–C*_(Å)	1.521	1.526	1.522
	r_*C–H*_(Å)	1.086	1.092	1.089
	YHĈC(deg)	111.4	111.23	111.2
Asymptote (CH_3_)				
	r_*C–C*_(Å)	6.0	10.0	
	r_*C*__–__*H*_(Å)	1.074	1.078	1.079

a From left to right, CAS-SCF(10e,10o)//CAS(PT2)/ANO-L, and rDSD/aug-cc-pVTZ optimized structural parameters and respective experimental data.

**Figure 6:**
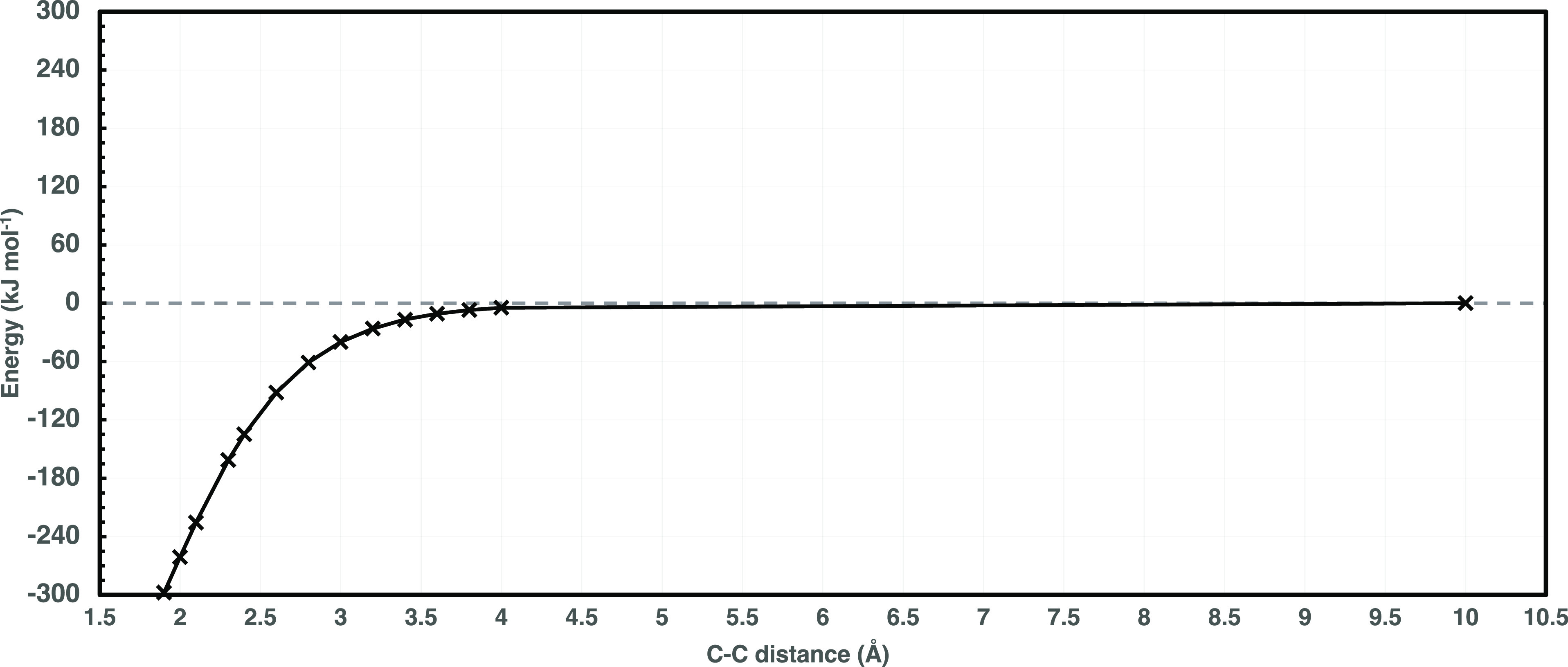
Ethane dissociation CAS-SCF(14e,14o)//CAS(PT2)/aug-cc-pVQZ relative potential energy profile (kJ mol^–1^) vs. C–C distance (Å). Stationary points were obtained by full geometry optimizations at the revDSD-PBEP86-D3(BJ)/aug-cc-pVTZ level of theory. Dashed horizontal line for reference zero energy value.

The rDSD model confirms its effectiveness and reliability. In detail, the C–C and C–H bond lengths of ethane are overestimated by 0.003 and 0.004 Å, respectively,whereas the H–Ĉ–C angle is in nearly exact agreement with the experiment. The CAS-SCF(10e,10o)//CAS(PT2)/ANO-L model^197^ delivers comparable results for bond lengths (overestimation of 0.001 and 0.003 Å for C–C and C–H bonds, respectively) but overestimates the H–Ĉ–C angle by 0.2°. Finally, the CH bond length of the methyl radical is underestimated by 0.005 and 0.001 Å at the CAS-SCF//CAS(PT2)(10e,10o)/ANO-L and rDSD level, respectively. In view of the remarkable agreement with experiment,^198^ the cheaper rDSD model is employed for performing the relaxed scan computations as a function of the C–C distance. Single-point CAS(PT2)//CAS-SCF/aug-cc-pVQZ energy evaluations at those geometries provide the reference potential, which is shown in Figure 6.

A comparison between this reference potential and the one employed by Klippenstein et al. in a very successful evaluation of rate constants^199^ is shown in Table 5. In general terms, a slightly more attractive potential is obtained in the present work, but the differences (average value of 1.1 kJ mol^–1^) are quite limited in view of the different geometries (rDSD vs B3LYP/6-31G*) and multireference model [CAS(PT2)/aug-cc-pVQZ vs CAS+1 + 2/aug-cc-pVTZ including also the Davidson correction].

**Table 5:** Ethane Dissociation TS Region Relative Energies

	relative energies (kJ mol^–1^)		
distance (Å)	ref^199^	present work	δ*E*
3.0	–38.2	–39.9	–1.7
3.2	–24.7	–25.9	–1.2
3.4	–15.3	–16.4	–1.1
3.6	–9.8	–10.8	–1.0
3.8	–6.0	–7.0	–1.0
4.0	–3.9	–4.5	–0.6

### Rate Constants of CH_3_ + CH_3_

The high-pressure rate constants obtained using the reference potential discussed above and an MC sampling based on BLYP-D3(BJ)/6-31++G(d,p) computations are collected in Table 6.

**Table 6:** VRC–VTST Barrier-less Rate Constants (cm^3^ molecule^–1^ s^–1^, 1 atm) at the UBLYP-D3(BJ)/6-31++G(d,p) Level of Theory^a^

	global rate constants	
*T*	expt^135^	uBLYP-D3(BJ)/6-31++G(d,p)
300	5.98 × 10^–^^11^	2.58 × 10^–^^11^
400	4.98 × 10^–^^11^	2.28 × 10^–^^11^
500	4.22 × 10^–^^11^	2.04 × 10^–^^11^
600	3.64 × 10^–^^11^	1.85 × 10^–^^11^
700	3.19 × 10^–^^11^	1.72 × 10^–^^11^
800	2.84 × 10^–^^11^	1.63 × 10^–^^11^
900	2.55 × 10^–^^11^	1.56 × 10^–^^11^
1000	2.31 × 10^–^^11^	1.51 × 10^–^^11^
1100	2.11 × 10^–^^11^	1.48 × 10^–^^11^
1200	1.94 × 10^–^^11^	1.46 × 10^–^^11^
1300	1.80 × 10^–^^11^	1.44 × 10^–^^11^
1400	1.67 × 10^–^^11^	1.44 × 10^–^^11^
1500	1.56 × 10^–^^11^	1.44 × 10^–^^11^
1600	1.46 × 10^–^^11^	1.44 × 10^–^^11^
1700	1.38 × 10^–^^11^	1.44 × 10^–^^11^
1800	1.30 × 10^–^^11^	1.45 × 10^–^^11^
1900	1.23 × 10^–^^11^	1.46 × 10^–^^11^
2000	1.17 × 10^–^^11^	1.48 × 10^–^^11^

a Temperature was in K (first column).

Inspection of Table 6 shows that the computed rate constants are within a factor of 2 of the corresponding experimental values in the whole temperature range, with the agreement improving with increasing the temperature. As is well known, the reactive flux shows a short-range minimum (around a separation of 2.6 Å between the methyl fragments), which is called the inner TS and is determined by the balance of enthalpy and entropy effects. For high angular momenta, a second minimum appears (the so-called outer TS), which is dominated by centrifugal effects. The role of the inner TS increases increasing the temperature. On the other hand, at low temperatures, the outer TS plays the dominant role, and the corresponding density of states is ruled by the transitional motions, whose DFT description does not benefit from the corrective potential. It is thus not unexpected that the rate constant at low temperatures is less accurate than its counterpart at high temperatures. While further improvements are being investigated for the more accurate sampling of transitional degrees of freedom, the present results are, in our opinion, already remarkably accurate.

## Conclusions

The kinetics of gas-phase reactions with barrier-less entrance channels is of remarkable interest in several fields, including, inter alia, combustion processes, atmospheric chemistry, and astrochemistry. Despite considerable progress in the theoretical background, algorithms, and computer implementations, some aspects deserve, in our opinion, further efforts. In the present study, we offer a contribution in that direction by means of an enhanced, user-friendly tool for the accurate yet effective computation of reactive fluxes. In particular, the integration of different flavors of DFT and wave function methods provides an optimal compromise between cost and accuracy. Furthermore, an algorithm enforcing smooth transitions between small- and large-amplitude vibrations along the RP significantly improves the computed rate constants. While the implementation of further enhancements is already in progress in our laboratories (especially concerning the selection and effective employment of the most effective internal curvilinear coordinates), the present version of the proposed tool paves the way for systematic investigations of gas-phase reactions of current theoretical and experimental interest.

## Supporting Information Available

Additional data for the behavior of different density functionals in the barrier-less steps of both reactions and of different wave function composite schemes for the relative energies of the stationary points governing the H_2_S + Cl reaction (PDF)
