# Supplementary material for: Toward an Accurate Black-Box Tool for the Kinetics of Gas-Phase Reactions Involving Barrier-less Elementary Steps
Source: J Chem Theory Comput. 2023 Oct 26;19(21):7626–39. doi: 10.1021/acs.jctc.3c00857 (PMC10653117; doi:10.1021/acs.jctc.3c00857)
Supplement: Supplementary file 1 — ct3c00857_si_001.pdf [file ct3c00857_si_001.pdf]

Supporting Information: Toward an Accurate Black-Box Tool For the Kinetics of Barrier-less Reactions in the Gas-Phase

Luigi Crisci,† Silvia Di Grande,†,‡ Carlo Cavallotti,¶ and Vincenzo Barone*,†

†*Scuola Normale Superiore di Pisa, Piazza dei Cavalieri 7, I-56126 Pisa, Italy*

‡*Scuola Superiore Meridionale, Largo San Marcellino 10, I-80138 Napoli, Italy*

¶*Department of Chemistry, Materials and Chemical Engineering "G. Natta", Politecnico di Milano, I-20131, Milano, Italy*

E-mail: vincenzo.barone@sns.it

Table S1: Max, minimum and average absolute relative deviations for ethane corrective potential sampling. All computations were performed applying D3(BJ) semi-empirical corrections.

Level of theory	Max Relative Deviation	Min Relative Deviation	Average Relative Deviation
uB2PLYP/6-311++G(3df,2p)	55%	37%	47%
uB2PLYP/6-311+G(3df,2p)	55%	37%	47%
uB2PLYP/6-311G(3df,2p)	57%	20%	40%
uB2PLYP/6-311++G(d,p)	58%	38%	50%
uB2PLYP/6-311+G(d,p)	58%	38%	50%
uB2PLYP/6-311G(d,p)	65%	34%	45%
uB2PLYP/6-31++G(3df,2p)	55%	37%	46%
uB2PLYP/6-31+G(3df,2p)	55%	37%	46%
uB2PLYP/6-31G(3df,2p)	63%	13%	36%
uB2PLYP/6-31++G(d,p)	60%	38%	50%
uB2PLYP/6-31+G(d,p)	61%	38%	51%
uB2PLYP/6-31G(d,p)	73%	34%	45%
uB2PLYP/aug-cc-pVDZ	55%	37%	44%
uB2PLYP/aug-cc-pVQZ	53%	37%	46%
uB2PLYP/aug-cc-pVTZ	54%	37%	46%
uB3LYP/6-311++G(3df,2p)	43%	26%	33%
uB3LYP/6-311+G(3df,2p)	43%	25%	33%
uB3LYP/6-311G(3df,2p)	42%	4%	26%
uB3LYP/6-311++G(d,p)	42%	27%	33%
uB3LYP/6-311+G(d,p)	42%	26%	33%
uB3LYP/6-311G(d,p)	41%	4%	27%
uB3LYP/6-31++G(3df,2p)	42%	16%	31%
uB3LYP/6-31+G(3df,2p)	42%	18%	31%
uB3LYP/6-31G(3df,2p)	41%	2%	22%
uB3LYP/6-31++G(d,p)	42%	20%	32%
uB3LYP/6-31+G(d,p)	42%	22%	32%
uB3LYP/6-31G(d,p)	46%	14%	26%
uB3LYP/aug-cc-pVDZ	43%	16%	30%
uB3LYP/aug-cc-pVQZ	42%	24%	33%
uB3LYP/aug-cc-pVTZ	43%	24%	33%

Table S2: Max, minimum and average absolute relative deviations for ethane corrective potential sampling. All computations were performed applying D3(BJ) semi-empirical corrections.

Level of theory	Max Relative Deviation	Min Relative Deviation	Average Relative Deviation
uBLYP/6-311++G(3df,2p)	40%	1%	12%
uBLYP/6-311+G(3df,2p)	40%	1%	12%
uBLYP/6-311G(3df,2p)	38%	7%	19%
uBLYP/6-311++G(d,p)	39%	0%	12%
uBLYP/6-311+G(d,p)	39%	1%	12%
uBLYP/6-311G(d,p)	38%	5%	16%
uBLYP/6-31++G(3df,2p)	39%	2%	14%
uBLYP/6-31+G(3df,2p)	39%	2%	14%
uBLYP/6-31G(3df,2p)	37%	3%	20%
uBLYP/6-31++G(d,p)	39%	0%	12%
uBLYP/6-31+G(d,p)	39%	0%	12%
uBLYP/6-31G(d,p)	36%	4%	14%
uBLYP/aug-cc-pVDZ	40%	5%	15%
uBLYP/aug-cc-pVQZ	39%	1%	13%
uBLYP/aug-cc-pVTZ	40%	1%	13%
uCAM-b3lyp/6-311++G(3df,2p)	50%	35%	44%
uCAM-b3lyp/6-311+G(3df,2p)	50%	35%	44%
uCAM-b3lyp/6-311G(3df,2p)	51%	7%	37%
uCAM-b3lyp/6-311++G(d,p)	50%	35%	44%
uCAM-b3lyp/6-311+G(d,p)	50%	35%	44%
uCAM-b3lyp/6-311G(d,p)	51%	15%	38%
uCAM-b3lyp/6-31++G(3df,2p)	50%	25%	42%
uCAM-b3lyp/6-31+G(3df,2p)	50%	26%	43%
uCAM-b3lyp/6-31G(3df,2p)	54%	1%	31%
uCAM-b3lyp/6-31++G(d,p)	51%	29%	43%
uCAM-b3lyp/6-31+G(d,p)	51%	31%	43%
uCAM-b3lyp/6-31G(d,p)	56%	24%	37%
uCAM-b3lyp/aug-cc-pVDZ	50%	26%	41%
uCAM-b3lyp/aug-cc-pVQZ	50%	34%	44%
uCAM-b3lyp/aug-cc-pVTZ	50%	34%	44%

Table S3: Max, minimum and average absolute relative deviations for ethane corrective potential sampling.

Level of theory	Max Relative Deviation	Min Relative Deviation	Average Deviation
uM06-2X/6-311++G(3df,2p)	69%	6%	35%
uM06-2X/6-311+G(3df,2p)	68%	6%	35%
uM06-2X/6-311G(3df,2p)	50%	1%	36%
uM06-2X/6-311++G(d,p)	68%	6%	35%
uM06-2X/6-311+G(d,p)	68%	6%	35%
uM06-2X/6-311G(d,p)	49%	4%	35%
uM06-2X/6-31++G(3df,2p)	62%	7%	35%
uM06-2X/6-31+G(3df,2p)	61%	8%	35%
uM06-2X/6-31G(3df,2p)	49%	0%	31%
uM06-2X/6-31++G(d,p)	58%	9%	35%
uM06-2X/6-31+G(d,p)	56%	8%	34%
uM06-2X/6-31G(d,p)	50%	6%	28%
uM06-2X/aug-cc-pVDZ	64%	3%	35%
uM06-2X/aug-cc-pVQZ	69%	8%	36%
uM06-2X/aug-cc-pVTZ	73%	5%	37%
uM06/6-311++G(3df,2p)	41%	8%	27%
uM06/6-311+G(3df,2p)	41%	8%	27%
uM06/6-311G(3df,2p)	40%	3%	23%
uM06/6-311++G(d,p)	41%	8%	27%
uM06/6-311+G(d,p)	41%	8%	26%
uM06/6-311G(d,p)	40%	1%	22%
uM06/6-31++G(3df,2p)	41%	14%	29%
uM06/6-31+G(3df,2p)	41%	14%	29%
uM06/6-31++G(d,p)	64%	17%	30%
uM06/6-31+G(d,p)	40%	17%	31%
uM06/6-31G(d,p)	40%	14%	27%
uM06/aug-cc-pVDZ	70%	7%	25%
uM06/aug-cc-pVQZ	41%	19%	32%
uM06/aug-cc-pVTZ	41%	16%	31%

Table S4: Max, minimum and average absolute relative deviations for ethane corrective potential sampling (counterpoise corrections). All computations were performed applying D3(BJ) semi-empirical corrections.

Level of theory	Max relative deviation	Min relative Deviation	Average Relative Deviation
uB2PLYP/6-311++G(3df,2p)	55%	38%	49%
uB2PLYP/6-311+G(3df,2p)	55%	38%	49%
uB2PLYP/6-311G(3df,2p)	57%	37%	50%
uB2PLYP/6-311++G(d,p)	58%	39%	52%
uB2PLYP/6-311+G(d,p)	58%	39%	52%
uB2PLYP/6-311G(d,p)	65%	38%	54%
uB2PLYP/6-31++G(3df,2p)	55%	38%	49%
uB2PLYP/6-31+G(3df,2p)	55%	38%	49%
uB2PLYP/6-31G(3df,2p)	63%	36%	53%
uB2PLYP/6-31++G(d,p)	60%	40%	53%
uB2PLYP/6-31+G(d,p)	61%	40%	54%
uB2PLYP/6-31G(d,p)	73%	38%	58%
uB2PLYP/aug-cc-pVDZ	55%	39%	49%
uB2PLYP/aug-cc-pVQZ	53%	37%	47%
uB2PLYP/aug-cc-pVTZ	54%	37%	47%
uB3LYP/6-311++G(3df,2p)	43%	26%	33%
uB3LYP/6-311+G(3df,2p)	43%	26%	33%
uB3LYP/6-311G(3df,2p)	42%	30%	35%
uB3LYP/6-311++G(d,p)	42%	28%	34%
uB3LYP/6-311+G(d,p)	42%	28%	34%
uB3LYP/6-311G(d,p)	41%	30%	36%
uB3LYP/6-31++G(3df,2p)	42%	25%	33%
uB3LYP/6-31+G(3df,2p)	42%	25%	33%
uB3LYP/6-31G(3df,2p)	41%	30%	38%
uB3LYP/6-31++G(d,p)	42%	28%	34%
uB3LYP/6-31+G(d,p)	42%	28%	34%
uB3LYP/6-31G(d,p)	46%	30%	39%
uB3LYP/aug-cc-pVDZ	43%	25%	33%
uB3LYP/aug-cc-pVQZ	42%	25%	33%
uB3LYP/aug-cc-pVTZ	43%	26%	33%

Table S5: Max, minimum and average absolute relative deviations for ethane corrective potential sampling (counterpoise corrections). All computations were performed applying D3(BJ) semi-empirical corrections.

Level of theory	Max relative deviation	Min relative Deviation	Average Relative Deviation
uBLYP/6-311++G(3df,2p)	40%	0%	12%
uBLYP/6-311+G(3df,2p)	40%	0%	12%
uBLYP/6-311G(3df,2p)	38%	1%	13%
uBLYP/6-311++G(d,p)	39%	0%	11%
uBLYP/6-311+G(d,p)	39%	0%	11%
uBLYP/6-311G(d,p)	38%	1%	14%
uBLYP/6-31++G(3df,2p)	39%	0%	12%
uBLYP/6-31+G(3df,2p)	39%	0%	12%
uBLYP/6-31G(3df,2p)	37%	1%	16%
uBLYP/6-31++G(d,p)	39%	0%	11%
uBLYP/6-31+G(d,p)	39%	0%	11%
uBLYP/6-31G(d,p)	36%	1%	17%
uBLYP/aug-cc-pVDZ	40%	2%	13%
uBLYP/aug-cc-pVQZ	39%	1%	12%
uBLYP/aug-cc-pVTZ	40%	1%	12%
uCAM-b3lyp/6-311++G(3df,2p)	50%	36%	45%
uCAM-b3lyp/6-311+G(3df,2p)	50%	36%	45%
uCAM-b3lyp/6-311G(3df,2p)	51%	39%	46%
uCAM-b3lyp/6-311++G(d,p)	50%	36%	45%
uCAM-b3lyp/6-311+G(d,p)	50%	36%	45%
uCAM-b3lyp/6-311G(d,p)	51%	39%	47%
uCAM-b3lyp/6-31++G(3df,2p)	50%	35%	45%
uCAM-b3lyp/6-31+G(3df,2p)	50%	35%	45%
uCAM-b3lyp/6-31G(3df,2p)	54%	38%	48%
uCAM-b3lyp/6-31++G(d,p)	51%	37%	45%
uCAM-b3lyp/6-31+G(d,p)	51%	38%	45%
uCAM-b3lyp/6-31G(d,p)	56%	37%	50%
uCAM-b3lyp/aug-cc-pVDZ	50%	34%	45%
uCAM-b3lyp/aug-cc-pVQZ	50%	35%	45%
uCAM-b3lyp/aug-cc-pVTZ	50%	35%	45%

Table S6: Max, minimum and average absolute relative deviations for ethane corrective potential sampling (counterpoise corrections).

Level of theory	Max relative deviation	Min relative Deviation	Average Relative Deviation
uM06-2X/6-311++G(3df,2p)	69%	7%	36%
uM06-2X/6-311+G(3df,2p)	68%	7%	36%
uM06-2X/6-311G(3df,2p)	50%	5%	34%
uM06-2X/6-311++G(d,p)	68%	7%	36%
uM06-2X/6-311+G(d,p)	68%	8%	36%
uM06-2X/6-311G(d,p)	49%	4%	33%
uM06-2X/6-31++G(3df,2p)	62%	9%	35%
uM06-2X/6-31+G(3df,2p)	61%	9%	35%
uM06-2X/6-31G(3df,2p)	49%	2%	30%
uM06-2X/6-31++G(d,p)	58%	7%	34%
uM06-2X/6-31+G(d,p)	56%	6%	34%
uM06-2X/6-31G(d,p)	50%	6%	32%
uM06-2X/aug-cc-pVDZ	64%	7%	35%
uM06-2X/aug-cc-pVQZ	69%	10%	36%
uM06-2X/aug-cc-pVTZ	73%	7%	37%
uM06/6-311++G(3df,2p)	41%	11%	28%
uM06/6-311+G(3df,2p)	41%	10%	28%
uM06/6-311G(3df,2p)	40%	16%	31%
uM06/6-311++G(d,p)	41%	10%	28%
uM06/6-311+G(d,p)	41%	10%	28%
uM06/6-311G(d,p)	40%	15%	30%
uM06/6-31++G(3df,2p)	41%	16%	31%
uM06/6-31+G(3df,2p)	41%	16%	31%
uM06/6-31G(3df,2p)	64%	29%	37%
uM06/6-31++G(d,p)	40%	18%	32%
uM06/6-31+G(d,p)	40%	19%	32%
uM06/6-31G(d,p)	70%	33%	40%
uM06/aug-cc-pVDZ	41%	13%	28%
uM06/aug-cc-pVTZ	41%	19%	32%
uM06/aug-cc-pVQZ	42%	21%	33%

Table S7: CCSD(T) relative energy ($kJ\,mol^{-1}$) for H_2S + Cl reaction employing different basis sets.

	Reactants	RW	TS	PW	Products
Basis set	H_2S + Cl	$H_2S \cdots$ Cl	HS $\cdots$ H $\cdots$ Cl	HS $\cdots$ HCl	HS + HCl
cc-pVTZ	0.00	-28.47	7.68	-56.06	-44.89
cc-pVQZ	0.00	-37.62	1.29	-58.21	-46.35
cc-pV5Z	0.00	-41.30	-0.82	-59.11	-46.96
cc-pV6Z	0.00	-42.76	-1.51	-59.45	-47.19
cc-pV(T+d)Z	0.00	-29.62	7.90	-55.95	-44.95
cc-pV(Q+d)Z	0.00	-38.33	1.38	-58.23	-46.46
cc-pV(5+d)Z	0.00	-41.58	-0.77	-59.18	-47.08
cc-pV(6+d)Z	0.00	-42.85	-1.47	-59.44	-47.20
aug-cc-pVTZ	0.00	-37.13	0.97	-59.36	-46.07
aug-cc-pVQZ	0.00	-41.40	-1.61	-59.78	-46.89
aug-cc-pV5Z	0.00	-43.30	-2.20	-59.98	-47.30
aug-cc-pV6Z	0.00	-43.70	-2.15	-59.85	-47.34
aug-cc-pV(T+d)Z	0.00	-38.12	1.33	-59.18	-46.09
aug-cc-pV(Q+d)Z	0.00	-41.92	-1.36	-59.72	-46.99
aug-cc-pV(5+d)Z	0.00	-43.35	-2.11	-60.04	-47.42
aug-cc-pV(6+d)Z	0.00	-43.71	-2.08	-59.82	-47.33
cc-pVTZ-F12	0.00	-36.38	1.44	-56.99	-44.95
cc-pVQZ-F12	0.00	-41.31	-1.06	-58.91	-46.53
aug-cc-pVTZ-F12	0.00	-38.09	0.18	-57.63	-44.94
aug-cc-pVQZ-F12	0.00	-42.21	-1.77	-59.38	-46.69
cc-pVTZ-F12+d_{diff}	0.00	-36.95	1.16	-57.13	45.03
cc-pVQZ-F12+d_{diff}+f_{diff}	0.00	-41.75	-1.30	-59.03	-46.58
cc-pV(T,Q)Z	0.00	-44.30	-3.37	-59.79	-47.42
cc-pV(Q,5)Z	0.00	-45.16	-3.04	-60.06	-47.59
cc-pV(5,6)Z	0.00	-44.77	-2.46	-59.91	-47.50
cc-pV((T+d),(Q+d))Z	0.00	-44.68	-3.37	-59.89	-47.56
cc-pV((Q+d),(5+d))Z	0.00	-45.00	-3.03	-60.17	-47.73
cc-pV((5+d),(6+d))Z	0.00	-44.58	-2.43	-59.80	-47.37
aug-cc-pV(T,Q)Z	0.00	-44.52	-3.49	-60.08	-47.48
aug-cc-pV(Q,5)Z	0.00	-45.29	-2.82	-60.20	-47.74
aug-cc-pV(5,6)Z	0.00	-44.25	-2.09	-59.66	-47.39
aug-cc-pV((T+d),(Q+d))Z	0.00	-44.70	-3.33	-60.11	-47.66
aug-cc-pV(((Q+d),(5+d))Z	0.00	-44.85	-2.89	-60.38	-47.87
aug-cc-pV((5+d),(6+d))Z	0.00	-44.21	-2.05	-59.50	-47.21
cc-pV(T,Q)Z-F12	0.00	-44.91	-2.89	-60.30	-47.68
aug-cc-pV(T,Q)Z-F12	0.00	-45.21	-3.19	-60.66	-47.96
cc-pV(T+d_{diff},Q+d_{diff}+f_{diff})Z-F12	0.00	-45.26	-3.10	-60.41	-47.71

Table S8: CCSD(T)-F12b relative energy ($\mathrm{kJ\,mol^{-1}}$) for $H_2S + Cl$ reaction employing different basis sets.

	Reactants	RW	TS	PW	Products
Basis set	H_2S + Cl	$H_2S \cdots$ Cl	HS $\cdots$ H $\cdots$ Cl	HS $\cdots$ HCl	HS + HCl
cc-pVTZ	0.00	-38.90	0.03	-58.82	-46.43
cc-pVQZ	0.00	-42.17	-1.45	-59.20	-46.93
cc-pV5Z	0.00	-43.35	-2.05	-59.62	-47.26
cc-pV(T+d)Z	0.00	-39.03	0.29	-58.99	-46.61
cc-pV(Q+d)Z	0.00	-42.23	-1.30	-59.40	-47.13
cc-pV(5+d)Z	0.00	-43.48	-2.01	-59.73	-47.42
aug-cc-pVTZ	0.00	-42.74	-2.22	-59.73	-47.05
aug-cc-pVQZ	0.00	-44.01	-2.69	-59.84	-47.21
aug-cc-pV5Z	0.00	-44.30	-2.59	-59.96	-47.45
aug-cc-pV(T+d)Z	0.00	-42.84	-1.99	-59.98	-47.29
aug-cc-pV(Q+d)Z	0.00	-43.84	-2.31	-59.91	-47.40
aug-cc-pV(5+d)Z	0.00	-44.28	-2.57	-60.10	-47.59
cc-pVTZ-F12	0.00	-42.12	-1.77	-59.27	-46.92
cc-pVQZ-F12	0.00	-43.54	-2.13	-59.63	-47.25
aug-cc-pVTZ-F12	0.00	-42.92	-2.23	-59.52	-47.02
aug-cc-pVQZ-F12	0.00	-44.02	-2.41	-59.85	-47.35
cc-pVTZ-F12+d_{diff}	0.00	-42.52	-1.87	-59.35	-47.05
cc-pVQZ-F12+d_{diff}+f_{diff}	0.00	-43.82	-2.22	-59.69	-47.30
cc-pV(T,Q)Z	0.00	-44.55	-2.54	-59.47	-47.29
cc-pV(Q,5)Z	0.00	-44.58	-2.68	-60.05	-47.61
cc-pV((T+d),(Q+d))Z	0.00	-44.57	-2.46	-59.69	-47.52
cc-pV((Q+d),(5+d))Z	0.00	-44.79	-2.76	-60.08	-47.71
aug-cc-pV(T,Q)Z	0.00	-44.94	-3.04	-59.92	-47.33
aug-cc-pV(Q,5)Z	0.00	-44.60	-2.49	-60.09	-47.70
aug-cc-pV((T+d),(Q+d))Z	0.00	-44.58	-2.55	-59.86	-47.48
aug-cc-pV(((Q+d),(5+d))Z	0.00	-44.74	-2.84	-60.29	-47.80
cc-pV(T,Q)Z-F12	0.00	-44.57	-2.39	-59.90	-47.49
aug-cc-pV(T,Q)Z-F12	0.00	-44.82	-2.55	-60.09	-47.59
cc-pV(T+d_{diff},Q+d_{diff}+f_{diff})Z-F12	0.00	-44.77	-2.47	-59.95	-47.48

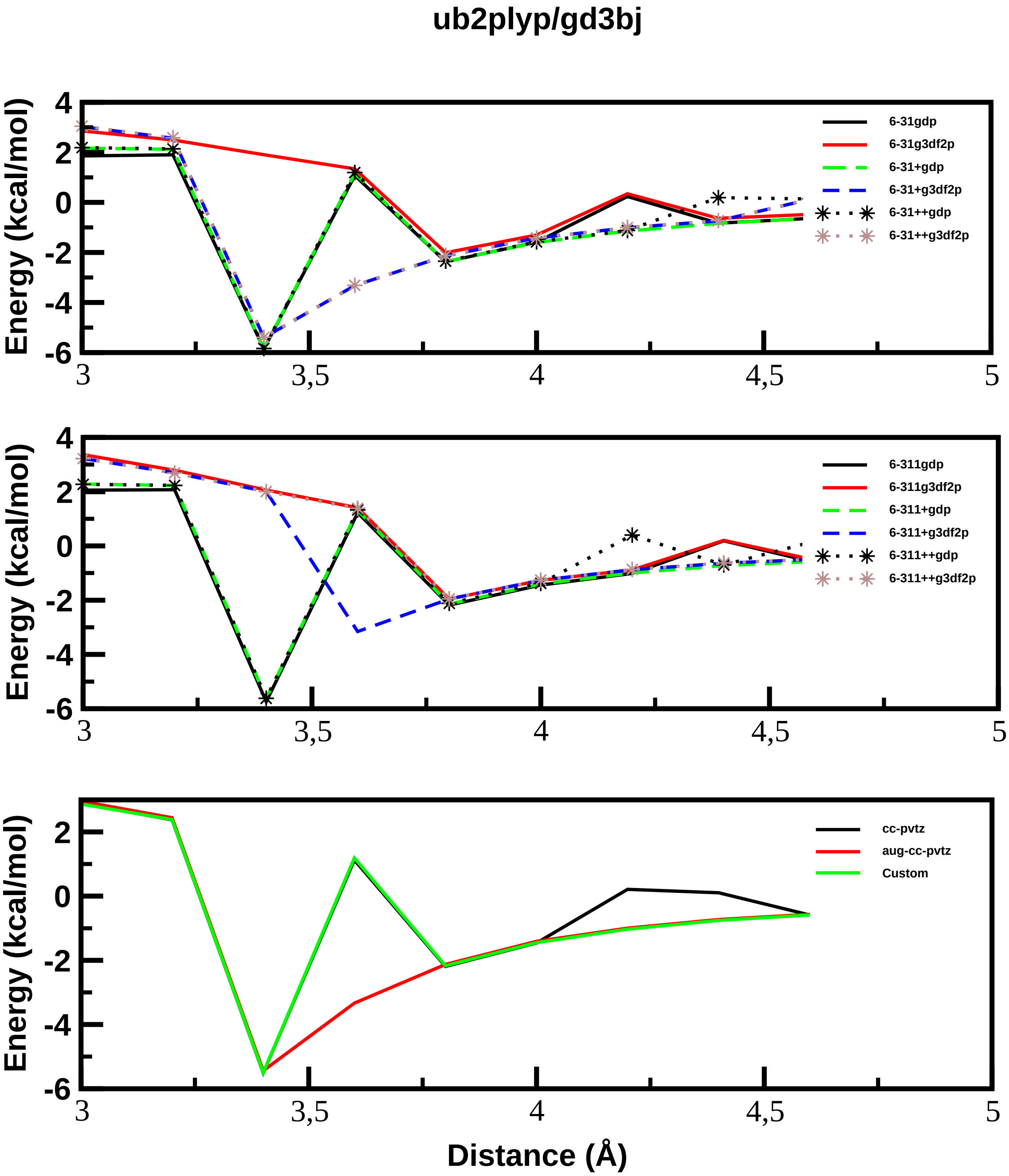

Figure S1: H_2S + Cl entrance channel uB2PLYP corrective potential (custom stands for jun-cc-pV(T+d)Z) (1 $\mathrm{kcal\,mol^{-1}} = 4.184\ \mathrm{kJ\,mol^{-1}}$)

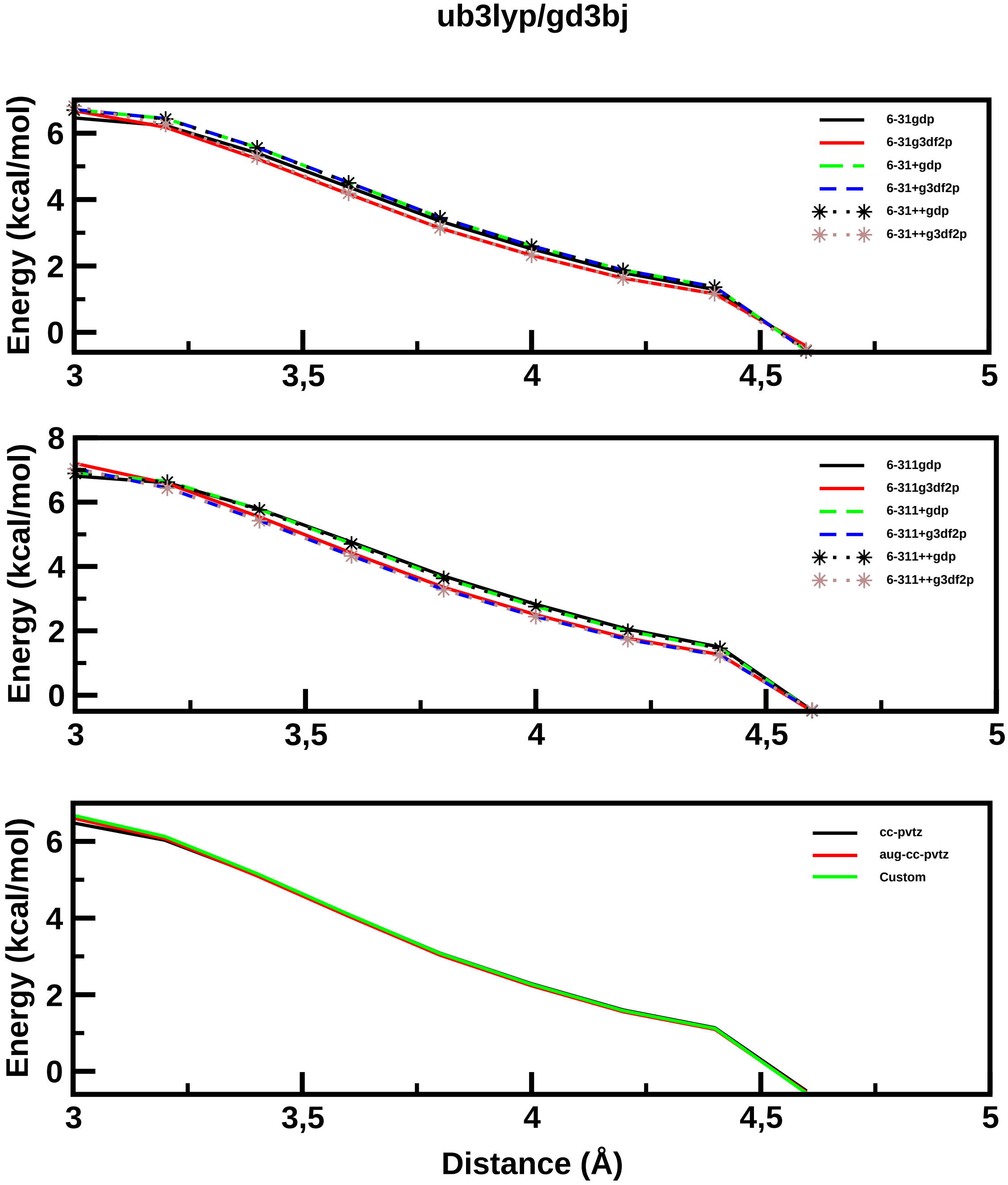

Figure S2: $H_2S + Cl$ entrance channel uB3LYP corrective potential (custom stands for jun-cc-pV(T+d)Z) in $\mathrm{kcal\,mol^{-1}}$ ($1\ \mathrm{kcal\,mol^{-1}} = 4.184\ \mathrm{kJ\,mol^{-1}}$)

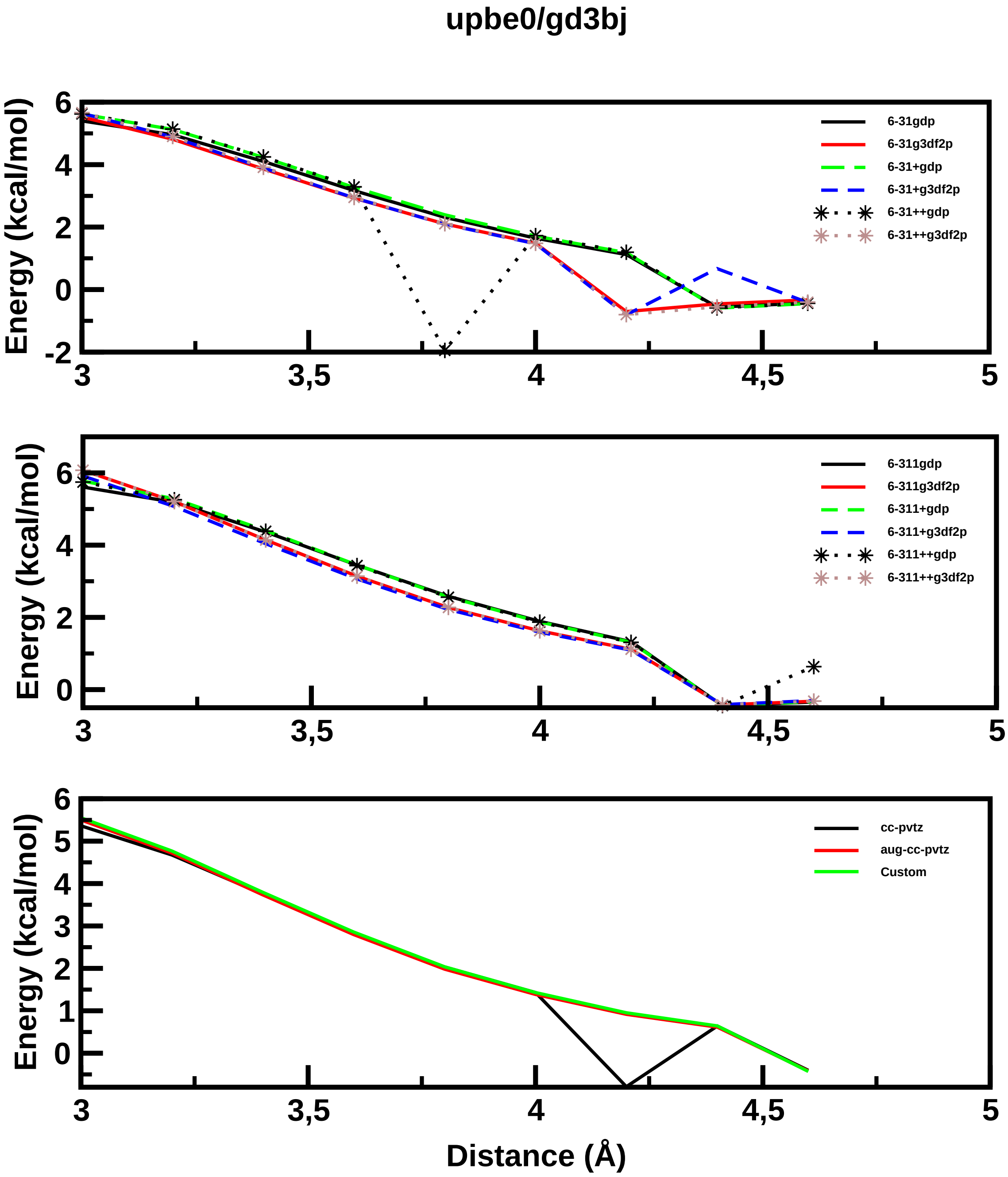

Figure S3: H_2S + Cl entrance channel uPBE0 corrective potential (custom stands for jun-cc-pV(T+d)Z) (1 $\mathrm{kcal\,mol^{-1}} = 4.184\ \mathrm{kJ\,mol^{-1}}$)

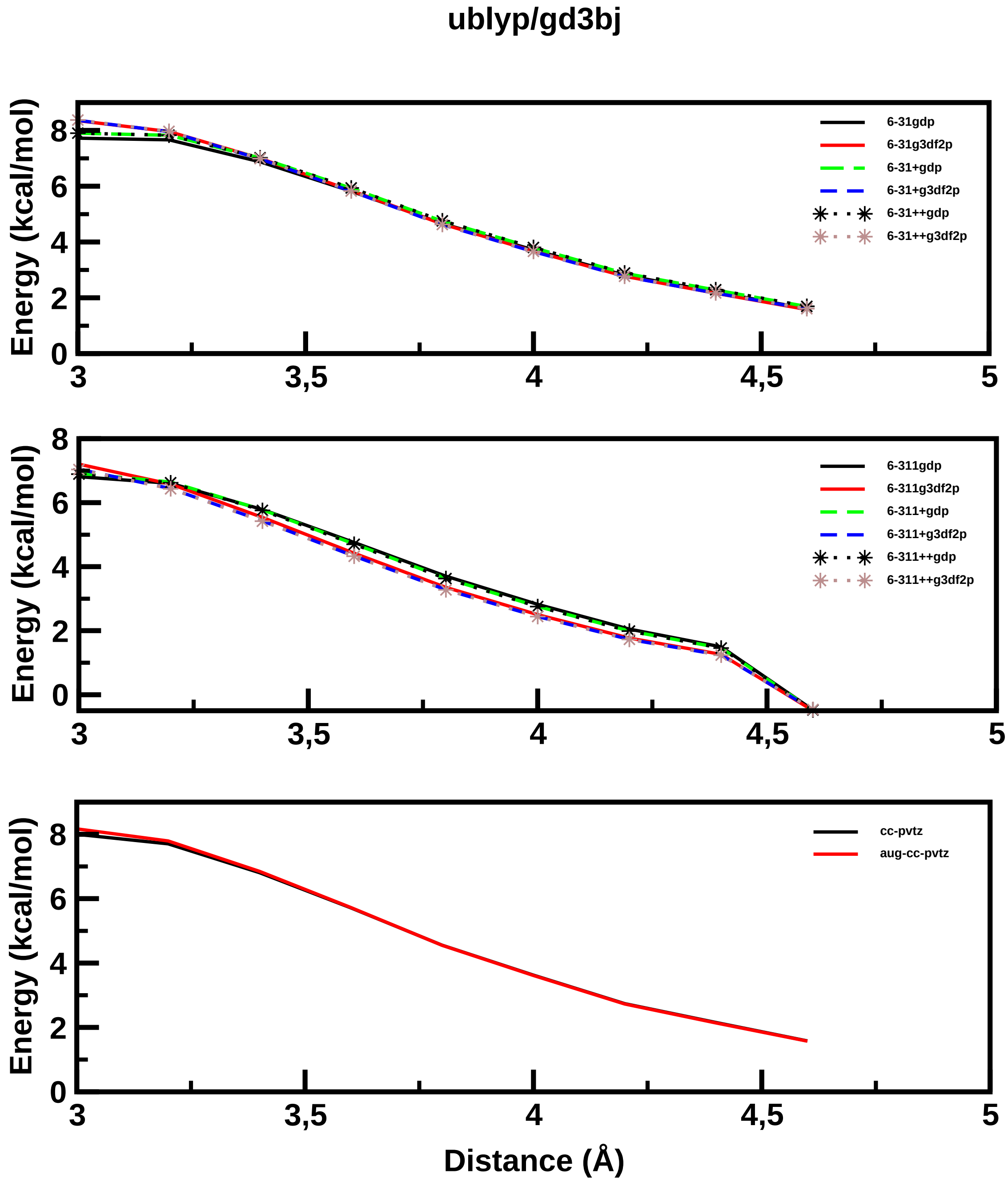

Figure S4: H_2S + Cl entrance channel uBLYP corrective potential (1 $\mathrm{kcal\,mol^{-1}}$ = 4.184 $\mathrm{kJ\,mol^{-1}}$)

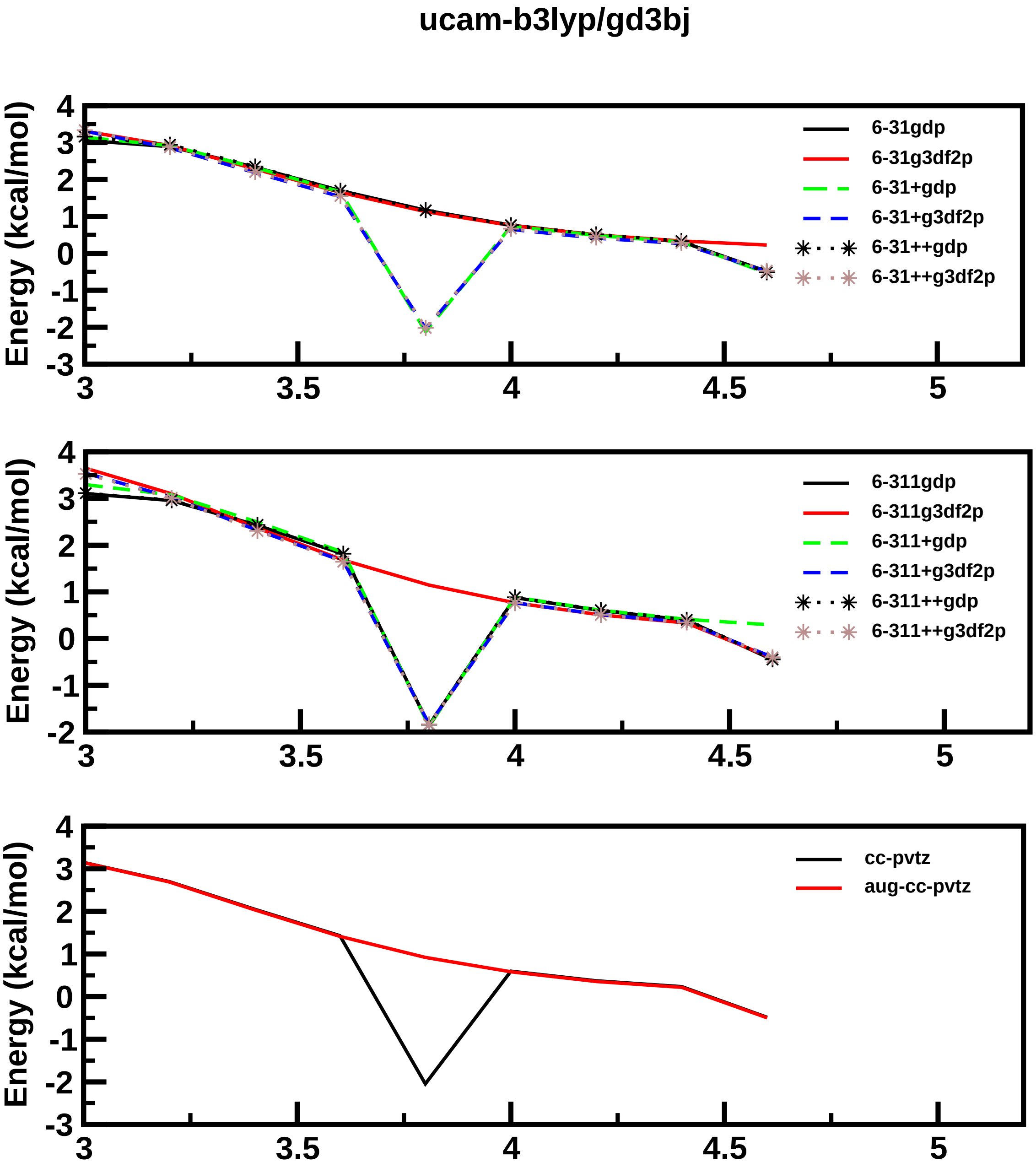

Figure S5: $H_2S + Cl$ entrance channel uCAM-B3LYP corrective potential ($1\ \mathrm{kcal\,mol^{-1}} = 4.184\ \mathrm{kJ\,mol^{-1}}$)

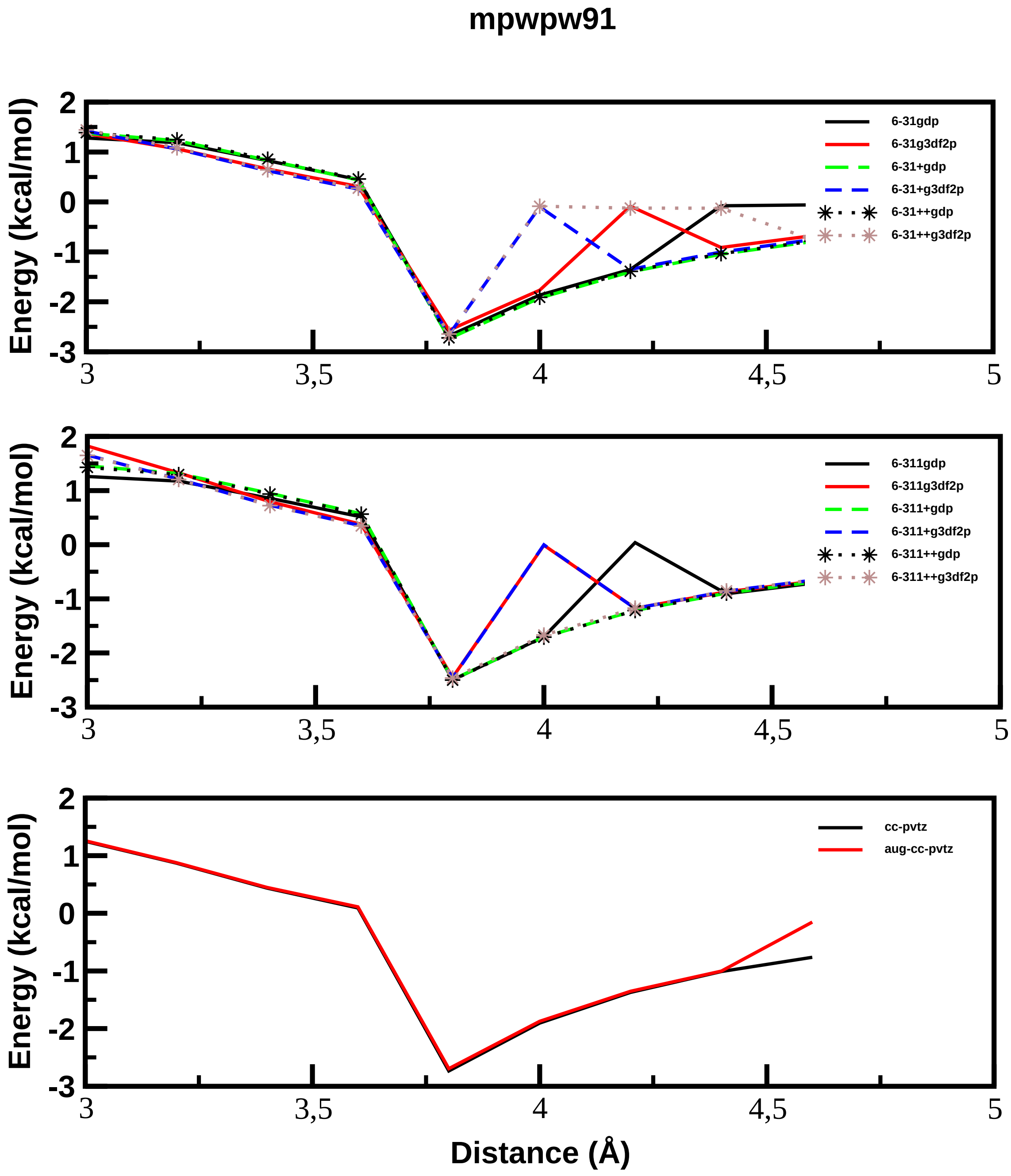

Figure S6: $H_2S + Cl$ entrance channel umPWPW91 corrective potential (1 kcal mol^{-1} = 4.184 kJ mol^{-1})

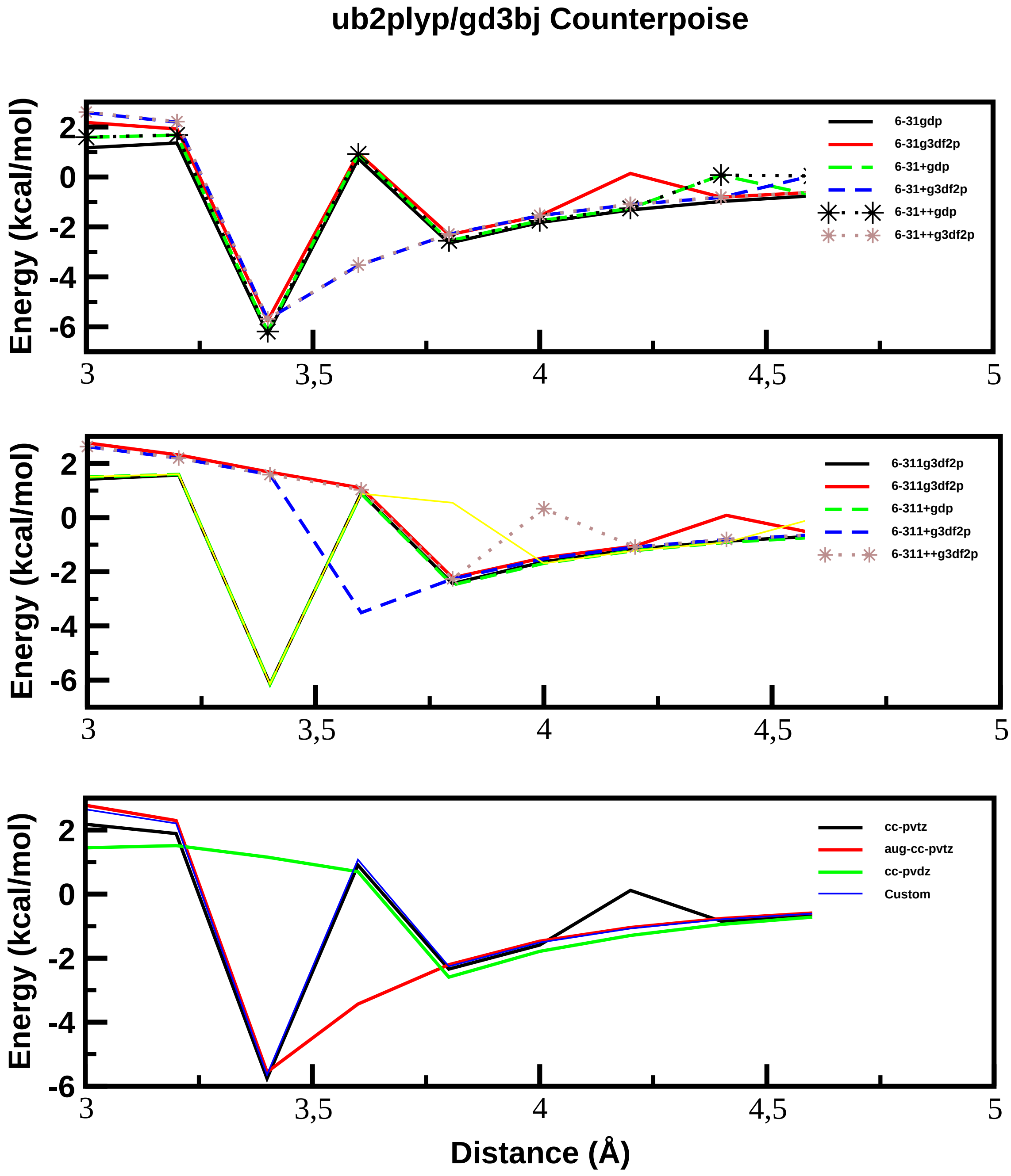

Figure S7: $H_2S + Cl$ entrance channel B2PLYP (counterpoise corrected) corrective potential (custom stands for jun-cc-pV(T+d)Z) (1 $\mathrm{kcal\,mol^{-1}} = 4.184\ \mathrm{kJ\,mol^{-1}}$)

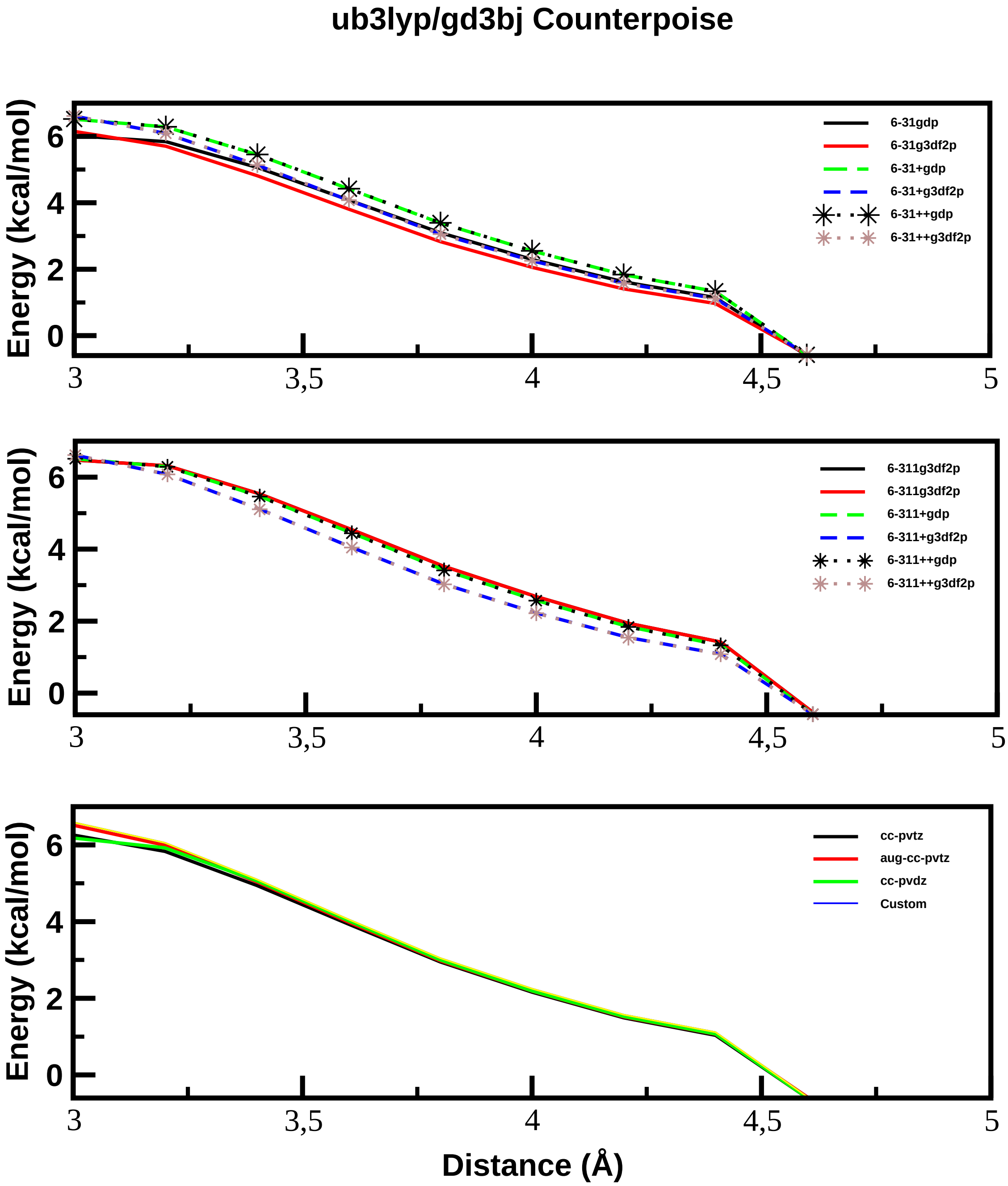

Figure S8: $H_2S + Cl$ entrance channel uB3LYP (counterpoise corrected) corrective potential (custom stands for jun-cc-pV(T+d)Z) (1 $kcal\,mol^{-1} = 4.184\ kJ\,mol^{-1}$)

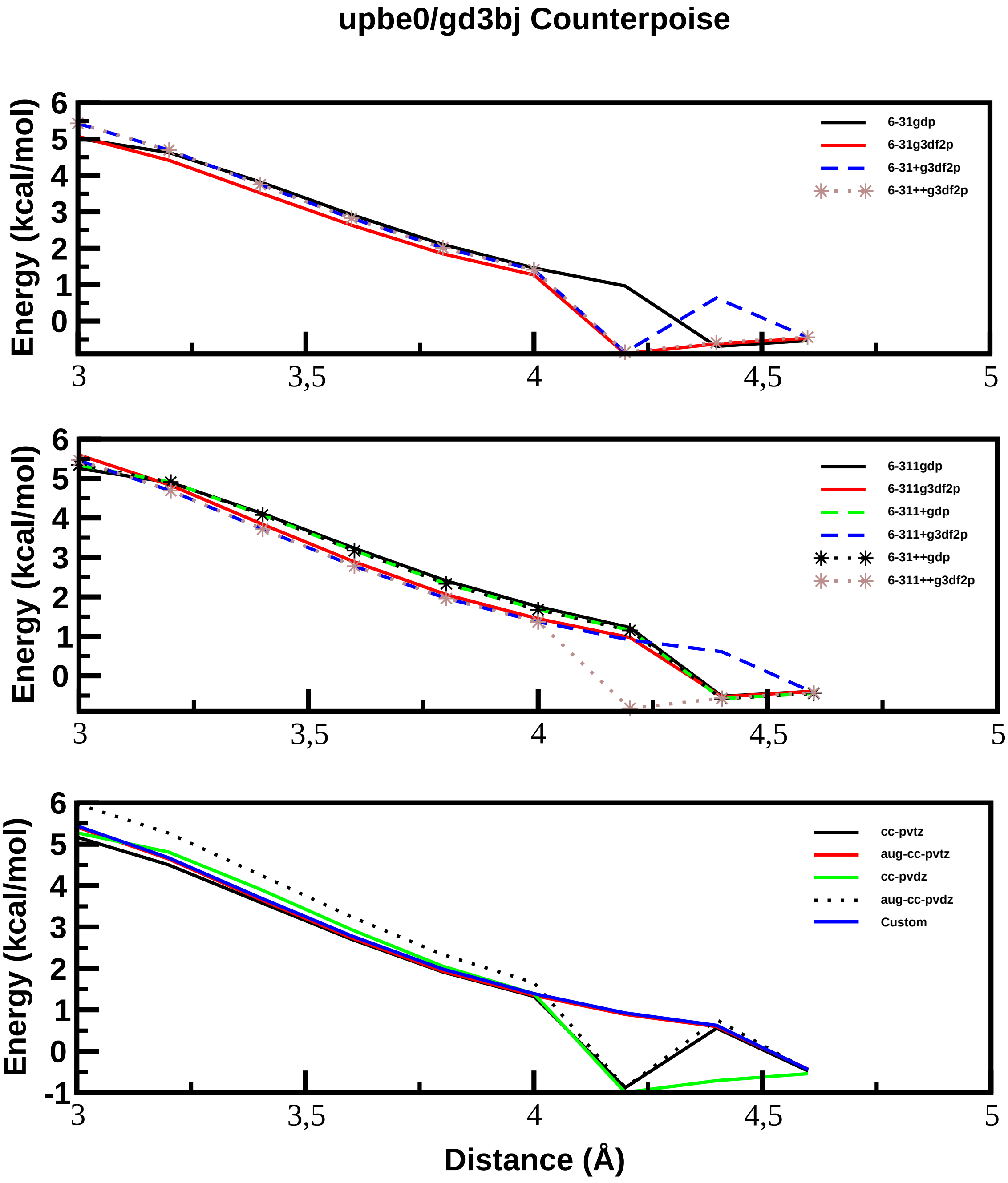

Figure S9: $H_2S + Cl$ entrance channel uPBE0 (counterpoise corrected) corrective potential (custom stands for jun-cc-pV(T+d)Z) (1 $\mathrm{kcal\,mol^{-1}} = 4.184\ \mathrm{kJ\,mol^{-1}}$)

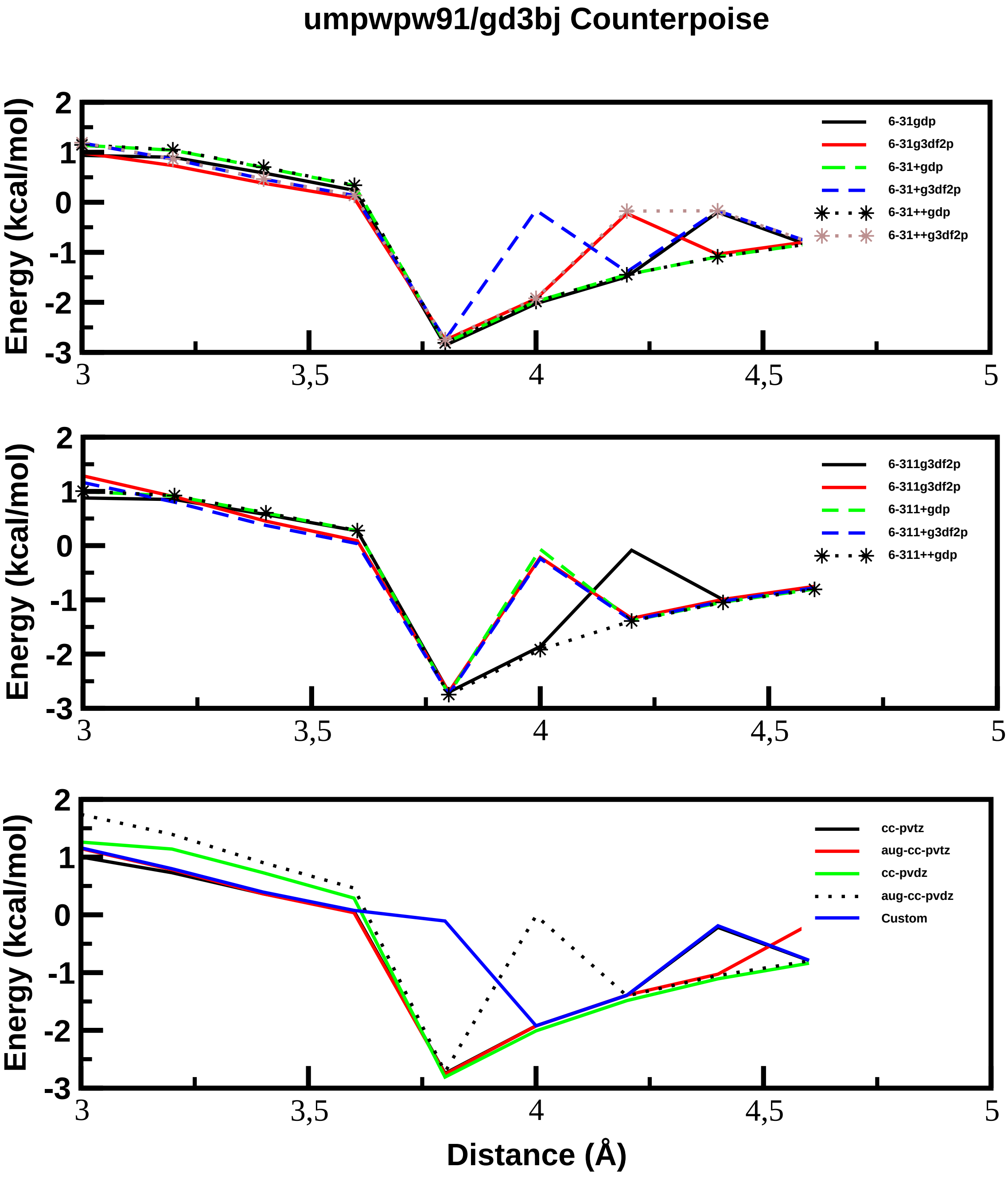

Figure S10: H_2S + Cl entrance channel umPWPW91 (counterpoise corrected) corrective potential (custom stands for jun-cc-pV(T+d)Z) (1 $\mathrm{kcal\,mol^{-1}} = 4.184\ \mathrm{kJ\,mol^{-1}}$)

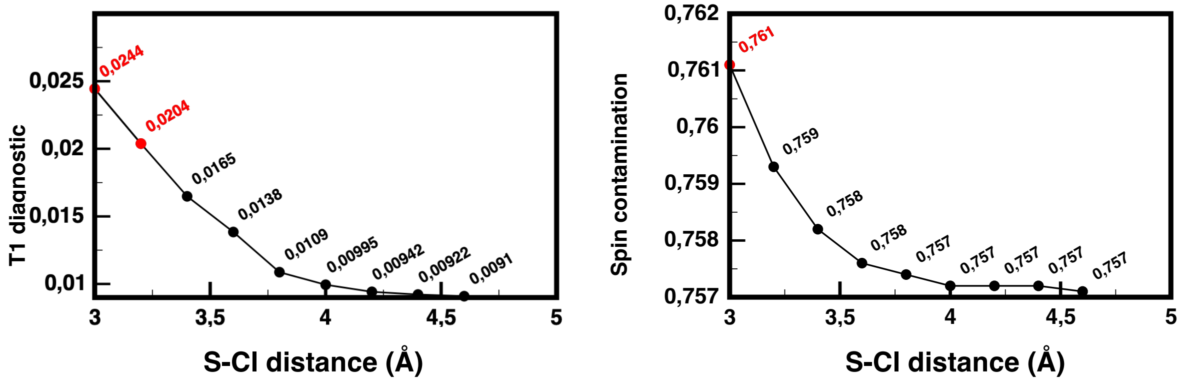

Figure S11: T1 diagnostic (left) and spin contamination (right) in the entrance channel of $H_2S + Cl$
